# Effect of Disparity in Self Dispersion Interactions on Phase Behaviors of Molten A-b-B Diblock Copolymers

**DOI:** 10.3390/polym15010030

**Published:** 2022-12-21

**Authors:** Xinyue Zhang, Mingge Zhao, Junhan Cho

**Affiliations:** Department of Polymer Science & Engineering, Dankook University, 152 Jukjeon-ro, Suji-gu, Yongin, Gyeonggi-do 16890, Republic of Korea

**Keywords:** diblock copolymer, Landau analysis, weak segregation regime, upper order-disorder transition, lower disorder-order transition, barotropicity, baroplasticity

## Abstract

Phase behaviors of molten A-b-B diblock copolymers with disparity in self dispersion interactions are revisited here. A free energy functional is obtained for the corresponding Gaussian copolymers under the influence of effective interactions originating in the localized excess equation of state. The Landau free energy expansion is then formulated as a series in powers of A and B density fluctuations up to 4th order. An alternative and equivalent Landau energy is also provided through the transformation of the order parameters to the fluctuations in block density difference and free volume fraction. The effective Flory *χ* is elicited from its quadratic term as the sum of the conventional enthalpic $\chi_{H}$ and the entropic $\chi_{S}$ that is related to energetic asymmetry mediated by copolymer bulk modulus. It is shown that the cubic term is balanced with Gaussian cubic vertex coefficients in corporation with energetics to yield a critical point at a composition rich in a component with stronger self interactions. The full phase diagrams with classical mesophases are given for the copolymers exhibiting ordering upon cooling and also for others revealing ordering reversely upon heating. These contrasting temperature responses, along with the skewness of phase boundaries, are discussed in relation to $\chi_{H}$ and $\chi_{S}$. The pressure dependence of their ordering transitions is either barotropic or baroplastic; or anomalously exhibits anomalously both at different stages. These actions are all explained by the opposite responses of $\chi_{H}$ and $\chi_{S}$ to pressure.

## 1. Introduction

Block copolymers have been of great importance for the past several decades because of their self-assembly into arrays of ordered nanoscopic structures such as lamellae, hexagonally packed cylinders, body-centered cubic spheres, double gyroids, other network structures, and Frank-Kasper phases [1,2,3,4]. Block copolymers are used in diverse areas and applications such as elastomers, surface modifiers, blend compatibilizers, and templates for directing structured materials towards data storage, nanolithography, and nanopattern transfer [5,6,7,8,9]. Block copolymers in selective solvents can be useful for drug delivery, cancer theranostics, nanoreactors, and stimuli-responsive materials [10,11].

It is well known from phenomenological studies on the corresponding incompressible copolymer systems that their phase behaviors are to be determined by the total number of monomers or chain size *N*, the component volume fractions *ϕ*, and the effective Flory interaction parameter *χ* [12]. However, the copolymer behaviors are considered to be much more complicated than the simple incompressible picture. It is typical that block copolymers exhibit ordering upon cooling, which is referred to as the upper order-disorder transition (UODT) [3,13]. Ordering of block copolymers upon heating has also been found, which is referred to as the lower disorder-order transition (LDOT) [14,15,16,17,18,19]. Some copolymers have been shown to reveal immiscibility loops [20,21,22,23] with both LDOT and UODT. These two types of temperature dependences of the ordering behaviors are driven by different mechanisms. The UODT has an enthalpic origin because it is driven by unfavorable energetics. On the contrary, the LDOT is of an entropic origin that is divided in three-fold ways [24]. Firstly, for copolymers with directional interactions between different monomers, there is entropic penalty in forming such directional pairs. Thus, increase in temperature allows those pairs less to phase separate less [25,26,27]. Secondly, the disparities in self dispersion interactions or compressibilities between component blocks lead to phase separation to gain more entropy through volume increase [24]. Thirdly, some polymer mixtures without directional interactions or compressibility differences exhibit phase separation because of entropic penalty arisen by asymmetry in monomer structures [28,29,30,31].

Block copolymers exhibiting either UODT or LDOT respond to pressure in two different ways. Firstly, their ordered region is enlarged upon pressurization, which is referred to as barotropicity. The unfavorable energetics are augmented by pressurization as a result of the densification of such interactions. Many UODT-type block copolymers such as polystyrene-b-polybutadiene (PS-b-PBD) and PS-b-polyisoprene (PS-b-PI) fall into this category in their responses to pressure [32,33,34,35,36]. Some strongly interacting LDOT-type mixtures exhibits barotropicity due to this densification effect [37]. The transition temperatures change typically by ~20 K over 100 MPa in the absolute sense. Secondly, the ordered region is shrunken upon pressurization, which is observed for some copolymers with substantial disparities in their compressibilities. This phenomenon is referred to as baroplasticity [35,38,39]. Some UODT-type copolymers such as PS-b-poly(n-hexyl methacrylate) (PS-*b*-PnHMA) [35] and PS-b-poly(ethyl hexly acrylate) (PS-b-PEHA) [40] are baroplastic. LDOT and loop-type block copolymers from PS and ethyl to n-pentyl polymethacrylates also exhibit this property [21,35,39]. The change in transition temperatures varies from ten to several hundred kelvin over 100 MPa in the absolute sense.

Over the years, we have sequentially developed sequentially the random-phase approximation theory [41,42,43], Landau analysis [44,45,46], and self-consistent field theory [47,48,49] for A-b-B block copolymers of all possible types exhibiting UODT, LDOT, barotropicity, and baroplasticity. Narrowing our attention down to Landau approach, the Landau free energy was first obtained as a series in powers of two order parameters, which are A and B density fluctuations, in a direct way [44]. Later in a separate study, an alternative Landau free energy was formulated through the transformation of order parameters [45,46]. The copolymer, with equal self-dispersion interactions for A and B blocks, reveals it is Landau free energy mathematically identical to that of the incompressible counterpart by Leibler. However, an effective Flory χ is shown to carry molecular parameters. Therefore, the symmetric copolymer exhibits a critical point (CP) that is pressure dependent [45,46]. It was argued that the copolymer with disparity in self dispersion interactions yields its Landau energy possessing the nonvanishing and negative cubic term, and the second-order transition is nullified even at the symmetric composition [44,46]. This energetic disparity gives asymmetry in densities or average intermonomer distances for different block domains. The notion that the copolymer phase transition is fully of first order seemed to be in harmony with other known facts. For the liquid-solid transition and isotropic-nematic transition in liquid crystals, their Landau free energy expansions usually possess nonvanishing cubic vertex coefficients [50]. These transitions are only of first order. Here, we revisit the phase behaviors of molten A-b-B diblock copolymers in the weak segregation regime. In the course of formulating the Landau free energy, it is understood that the effective cubic order term is more intricate than previously studied. It is shown that our Landau energy with the deepened conception resurrects the CP, whereas its location is dependent on the disparity in self dispersion interactions. The Landau free energy is derived in two different ways; one is in a direct way with the two order parameters, and the other is through the transformation of the order parameters. Using these two equivalent free energies, the theoretical calculation of the copolymer phase behaviors and transitions are to be compared with experimental results.

## 2. Theory

### 2.1. Free Energy Density in the Bulk State

Our system of interest is A-b-B diblock copolymer chains made of A and B monomers in volume *V*. There are $n_{c}$ such chains, where each *j*-block possesses $N_{j}$ tangent spheres having the identical diameter *σ*. The close packed volume of *j*-blocks in the system is given as $V_{j}=n_{c}N_{j}v^{*}$, where $v^{*}=\pi \sigma^{3}/6$ is the monomer volume. Then, the close packed volume fraction of *j*-block is given as $\phi_{j}=V_{j}/\sum V_{k}$$=N_{j}/N_{c}$, where $N_{c}=$$N_{A}+N_{B}$ is the copolymer chain size. The overall packing density *η* is given by $\eta =\sum V_{j}/V$, and the packing density of *j*-block is equal to $\eta_{j}=\phi_{j}\eta$.

The Helmholtz free energy *A* of the copolymer melt is given as the sum of ideal $A_{id}$ and non-ideal $A_{ni}$ as $A=A_{id}+A_{ni}$ [41,46,47]. The former $A_{id}$ is given below:

(1) $$\frac{\beta A_{id}v*}{V}=\frac{\eta }{N_{c}}\ln\frac{\eta K}{N_{c}}$$

where $\beta =1/k_{B}T$ as usual, and *K* is the molecular constant that does not affect any thermodynamic properties. The latter $A_{ni}$ is subdivided into $A_{ni}=A_{HSC}+U_{nb}$, where $A_{HSC}$ implies the excluded volume contribution by hard sphere chains, and $U_{nb}$ represents dispersion (van der Waals) interaction energy between nonbonded monomers. The first contribution $A_{HSC}$ is formulated from Baxter’s integral equation theory for adhesive hard spheres under Chiew’s connectivity constraint [51,52,53]. Mathematically stated,

(2) $$\frac{\beta A_{HSC}v*}{V}=\frac{3}{2}\left[\frac{\eta }{{\left(1-\eta \right)}^{2}}-\left(1-\frac{1}{N_{c}}\right)\frac{\eta }{1-\eta }\right]-\frac{\eta }{N_{c}}\left[\ln\left(1-\eta \right)+\frac{3}{2}\right]$$

The second contribution $U_{nb}$ is obtained from the Bethe-Peierls-type mean-field energy [54] of locally packed nearest-neighbors around a chosen monomer. There are AA, AB, and BB pairs, whose contact energies are represented by ${\bar{\varepsilon }}_{AA}$, ${\bar{\varepsilon }}_{AB}$, and ${\bar{\varepsilon }}_{BB}$, respectively. Then, $U_{nb}$ can be written as

(3) $$\frac{\beta U_{nb}v*}{V}=\frac{1}{2}\cdot \beta \cdot \sum_{ij}\phi_{i}\phi_{j}{\bar{\varepsilon }}_{ij}\cdot u(\eta )\cdot \eta =\frac{1}{2}\cdot \beta \cdot \sum_{ij}\eta_{i}\eta_{j}{\bar{\varepsilon }}_{ij}\cdot \frac{u(\eta )}{\eta }$$

The density dependence of $U_{nb}$ is determined by u(η)=$4[{\left(\gamma /C\right)}^{4}\eta^{4}-{\left(\gamma /C\right)}^{2}\eta^{2}]$ with $\gamma =1/\sqrt{2}$ and C=π/6. We denote the free energy *A* per unit volume as a≡A/V, and its nonideal part as $a_{ni}\equiv A_{ni}/V$.

### 2.2. Series Expansion of Free Energy Functional

The free energy density functional for an inhomogeneous A-B diblock copolymer melt is written in general as [47]

(4) $$\frac{\beta A_{inh}v^{*}}{V}=\frac{\eta }{N_{c}}\ln\frac{\eta K}{N_{c}}-\frac{\eta }{N_{c}}\ln\left(\frac{1}{V}\int_{}^{}d\vec{r}\cdot q(\vec{r},1)\right)+\frac{1}{V}\left(\int_{}^{}d\vec{r}\cdot \beta a_{ni}(\vec{r})v*-\sum_{j}\int_{}^{}d\vec{r}\cdot i\omega_{j}(\vec{r})\cdot \eta_{j}(\vec{r})\right)$$

where $a_{ni}\left(\vec{r}\right)$ is the localized $a_{ni}$ to give the effective short-ranged interactions. The function $\omega_{j}(\vec{r})$ indicates the external potential conjugate to the local j-density $\eta_{j}(\vec{r})$. In Equation (4), *q* is the end-segment distribution function of Gaussian A-b-B chains subject to $\omega_{j}$s which transmits the influence of the local interactions to the chain conformations to describe microphase segregated state.

Fluctuations in various field variables are defined by $\Delta \eta_{j}(\vec{r})\equiv \eta_{j}(\vec{r})-\eta_{j}$ and $\Delta \omega_{j}(\vec{r})\equiv \omega_{j}(\vec{r})$, where the spatial average of $\omega_{j}$ is shifted to zero. Then, the logarithm of *Q* ($\equiv 1/V\cdot \int qd\vec{r}$) in Equation (4) can be expanded as a series in powers of $\omega_{j}$s up to 4th order as

(5) $$\ln Q=\ln\left[\frac{1}{V}\int_{}^{}d\vec{r}\cdot q(\vec{r},1)\right]=\ln\bar{Q}+\sum_{n=2}^{4}\frac{{\left(-1\right)}^{n}N_{c}}{n!V}\int \prod_{l=1}^{n}\frac{d{\vec{k}}_{l}}{{\left(2\pi \right)}^{3}}\cdot G_{i_{1},\ldots ,i_{n}}^{(n)0}({\vec{k}}_{1},\ldots ,{\vec{k}}_{n})\omega_{i_{1}}({\vec{k}}_{1})\ldots \omega_{i_{n}}({\vec{k}}_{n})$$

where $\bar{Q}$ is defined by $\bar{Q}=Q\left(\omega_{j}\to 0\right)$, and $G_{ij}^{(2)0}$, $G_{ijk}^{(3)0}$, and $G_{ijkl}^{(4)0}$ are the proper Gaussian correlation functions. It is common to replace $G_{ij}^{(2)0}$ with $S_{ij}^{0}$. Equation (5) is written in Fourier form with scattering vectors $\vec{k}$s. In our A-b-B copolymer system, $S_{AA}^{0}(\vec{k})=\eta N\cdot d_{1}(\phi_{A},x)$ is used for AA correlations with its gyration radius $R_{G}^{}$, where $d_{1}(\phi_{A},x)=2/x^{2}\cdot (e^{-\phi_{A}x}+\phi_{A}x-1)$ is the modified Debye function and $x\equiv k^{2}R_{G}^{2}$. Likewise, $S_{BB}^{0}(\vec{k})=\eta N\cdot d_{1}(1-\phi_{A},x)$ is used for BB correlations. The remaining AB correlations is described by $S_{AB}^{0}(\vec{k})=$$\eta N/2\cdot \left[d_{1}(1,x)-d_{1}(\phi_{A},x)-d_{1}(1-\phi_{A},x)\right]$.

Now, the free energy is written below as

(6) $$\begin{array}{l} \frac{\beta A_{inh}v^{*}}{V}\approx \frac{\eta }{N_{c}}\ln\frac{\eta K}{N_{c}}+\sum_{n=2}^{4}\frac{(-1{)}^{n}N_{c}}{n!V}\cdot \int \prod_{l=1}^{n}\frac{d{\vec{k}}_{l}}{(2\pi {)}^{3}}\cdot G_{i_{1},\ldots ,i_{n}}^{(n)0}\left({\vec{k}}_{1},\ldots ,{\vec{k}}_{n}\right)\omega_{i}\left({\vec{k}}_{1}\right)\ldots \omega_{i_{n}}\left({\vec{k}}_{n}\right)\\ +\frac{1}{V}\left(\beta {\bar{a}}_{ni}v^{*}V+\frac{1}{2}\sum_{i,j}\int \frac{d\vec{k}}{(2\pi {)}^{3}}\cdot \beta D_{ij}a\cdot v^{*}\Delta \eta_{i}(\vec{k})\Delta \eta_{j}(-\vec{k})\right)-\frac{1}{V}\left(\sum_{j}\int \frac{d\vec{k}}{(2\pi {)}^{3}}\cdot \omega_{j}(\vec{k})\cdot \Delta \eta_{j}(-\vec{k})\right) \end{array}$$

where $\bar{Q}$ is absorbed into *K* and ${\bar{a}}_{ni}$ indicates $a_{ni}$ in the homogeneous state. The symbol $D_{ij}a$ denotes the second-order derivatives of $a_{ni}$ as $D_{ij}a\equiv \partial^{2}a_{ni}/\partial \eta_{i}\partial \eta_{j}$ to give the effective local interactions in two-body level. For compressible systems, the Gaussian correlation functions are diluted by *η* because of free volume.

The Landau free energy is formulated from Equation (6) by replacing $\Delta \eta_{j}(\vec{k})$ and $\omega_{j}(\vec{k})$ with their ensemble averages. For simplicity, we will use the same symbols for their averages. To minimize the Landau free energy, it is required that $\delta \left(A_{inh}/V\right)/\delta \omega_{j}=0$, which yields the relations between $\omega_{j}(\vec{k})$s and $\Delta \eta_{j}(\vec{k})$s. The Landau free energy is then re-written as a series in powers of $\Delta \eta_{j}(\vec{k})$s as follows:

(7) $$\begin{array}{l} \frac{\beta A_{inh}v^{*}}{V}=\frac{\eta }{N_{c}}\ln\frac{\eta K}{N_{c}}+\beta {\bar{a}}_{ni}v^{*}+\frac{1}{2!V}\sum_{ij}\left[\int \frac{d{\vec{k}}_{1}}{(2\pi {)}^{3}}\frac{d{\vec{k}}_{2}}{(2\pi {)}^{3}}\Gamma_{ij}^{\left(2\right)}\left({\vec{k}}_{1},{\vec{k}}_{2}\right)\Delta \eta_{i}\left({\vec{k}}_{1}\right)\Delta \eta_{j}\left({\vec{k}}_{2}\right)\right]\\ +\frac{1}{3!V}\sum_{ijk}\left[\int \frac{d{\vec{k}}_{1}}{(2\pi {)}^{3}}\frac{d{\vec{k}}_{2}}{(2\pi {)}^{3}}\frac{d{\vec{k}}_{3}}{(2\pi {)}^{3}}\Gamma_{ijk}^{\left(3\right)}\left({\vec{k}}_{1},{\vec{k}}_{2},{\vec{k}}_{3}\right)\Delta \eta_{i}\left({\vec{k}}_{1}\right)\Delta \eta_{j}\left({\vec{k}}_{2}\right)\Delta \eta_{k}\left({\vec{k}}_{3}\right)\right]\\ +\frac{1}{4!V}\sum_{ijkl}\left[\int \frac{d{\vec{k}}_{1}}{(2\pi {)}^{3}}\frac{d{\vec{k}}_{2}}{(2\pi {)}^{3}}\frac{d{\vec{k}}_{3}}{(2\pi {)}^{3}}\frac{d{\vec{k}}_{4}}{(2\pi {)}^{3}}\Gamma_{ijkl}^{\left(4\right)}\left({\vec{k}}_{1},{\vec{k}}_{2},{\vec{k}}_{3},{\vec{k}}_{4}\right)\Delta \eta_{i}\left({\vec{k}}_{1}\right)\Delta \eta_{j}\left({\vec{k}}_{2}\right)\Delta \eta_{k}\left({\vec{k}}_{3}\right)\Delta \eta_{l}\left({\vec{k}}_{4}\right)\right]+O\left(\Delta \eta_{j}^{5}\right) \end{array}$$

The second-order vertex function $\Gamma_{ij}^{\left(2\right)}$ is identical to $S_{ij}^{-1}$, which is given by the sum of Gaussian $S_{ij}^{0-1}$ and effective interaction fields. The higher-order vertex functions are obtained as the combination of Gaussian correlation functions, which can be found elsewhere [12,44,46]. It should be recognized that all the vertex functions require $\sum k_{i}=0$.

### 2.3. Formulation of Landau Free Energy

#### 2.3.1. Method I: Direct Way

Owing to the covalent bonds between A and B blocks, A-b-B diblock copolymer melts exhibit phase separation only on a nanometer scale. These nanoscale mesophases are diverse, but here we consider only the classical ones such as 3-dimensional body-centered cubic spheres (BCC), 2-dimensional hexagonally packed cylinders (HEX), and 1-dimensional lamellae (LAM). The quadratic form of the free energy functional expansion yields the characteristic wavenumber $k^{*}$ at its minimum, which in turn gives the periodicity of the repeating structures with the domain size *D* as $D=2\pi /k^{*}$. These nanostructures are determined by *n* characteristic scattering vectors ${\vec{K}}_{j}$s, whose magnitudes are $\left|{\vec{K}}_{j}\right|=k^{*}$.

Lamellar mesophase possesses one base vector ${\vec{K}}_{1}=k^{*}\cdot \left(1,0,0\right)$ with *n* = 1. Meanwhile, HEX mesophase possesses three base vectors, ${\vec{K}}_{1}=k^{*}\cdot \left(1,0,0\right)$, ${\vec{K}}_{2}=k^{*}\cdot \left(-1/2,\sqrt{3}/2,0\right)$, ${\vec{K}}_{3}=k^{*}\cdot \left(-1/2,-\sqrt{3}/2,0\right)$ along with *n* = 3. The last BCC mesophase possesses six base vectors, ${\vec{K}}_{1}=k^{*}/\sqrt{2}\cdot \left(1,1,0\right)$, ${\vec{K}}_{2}=k^{*}/\sqrt{2}\cdot \left(-1,1,0\right)$, ${\vec{K}}_{3}=k^{*}/\sqrt{2}\cdot \left(0,1,1\right)$, ${\vec{K}}_{4}=k^{*}/\sqrt{2}\cdot \left(0,1,-1\right)$, ${\vec{K}}_{5}=k^{*}/\sqrt{2}\cdot \left(1,0,1\right)$, ${\vec{K}}_{6}=k^{*}/\sqrt{2}\cdot \left(1,0,-1\right)$, along with *n* = 6.

Following Leibler’s seminal analysis [12,44], the integral in Equation (7) is approximated to the finite sum of integrands at ${\vec{K}}_{j}$. Each $\Delta \eta_{i}(\pm {\vec{k}}_{1})$ is now treated as a plane wave with its amplitude $\left(1/\sqrt{n}\right)\varsigma_{j}$ and phase angle $\pm \varphi \left(i\right)$ as $\Delta \eta_{i}(\pm {\vec{k}}_{1})=\left(1/\sqrt{n}\right)\varsigma_{i}e^{\pm i\varphi \left(i\right)}$. The free energy expansion is greatly simplified to yield the following form as a series in powers of $\varsigma_{j}$s up to 4th order:

(8) $$\begin{array}{lll} \beta \Delta A & = & \left(\Gamma_{AA}^{}\varsigma_{A}^{2}-2\Gamma_{AB}^{}\varsigma_{A}^{}\varsigma_{B}^{}+\Gamma_{BB}^{}\varsigma_{B}^{2}\right)\\ & & -\left|\alpha_{AAA}\varsigma_{A}^{3}-3\alpha_{AAB}\varsigma_{A}^{2}\varsigma_{B}^{}+3\alpha_{ABB}\varsigma_{A}^{}\varsigma_{B}^{2}-\alpha_{BBB}\varsigma_{B}^{3}\right|+\delta_{ijkl}e^{-i\pi \cdot c_{B}\left(ijkl\right)}\cdot \varsigma_{i}^{}\varsigma_{j}^{}\varsigma_{k}^{}\varsigma_{l}^{} \end{array}$$

where the necessary treatment of the vertex coefficients of Equation (7) for the three mesophases is given in the Appendix A. In Equation (8), Einstein’s summation convention is used when necessary. It is seen that the permutation of indices of $\alpha_{AAB}$ and $\alpha_{ABB}$ yields the identical vertex function values. The cubic coefficients $\alpha_{ijk}$s for LAM, HEX, and BCC are given respectively as follows:

(9) $$\alpha_{ijk}^{LAM}=0;\;\alpha_{ijk}^{HEX}=\frac{12}{3!{\left(\sqrt{3}\right)}^{3}}\Gamma_{ijk}\left(1\right);\;\alpha_{ijk}^{BCC}=\frac{48}{3!{\left(\sqrt{6}\right)}^{3}}\Gamma_{ijk}\left(1\right)$$

In Equation (9), a number *h* is put into the bracket to indicate the relative angles between the three scattering vectors ${\vec{k}}_{1}$, ${\vec{k}}_{2}$, and ${\vec{k}}_{3}$, where its definition is $h\equiv {\left|{\vec{k}}_{1}+{\vec{k}}_{2}\right|}^{2}/{\left(k*\right)}^{2}$. Then, the right triangular arrangement of those vectors yields $\left|{\vec{k}}_{1}+{\vec{k}}_{2}\right|=\left|{\vec{k}}_{3}\right|$ and *h* = 1. The quartic coefficients $\delta_{ijkl}$s are obtained as

(10) $$\delta_{ijkl}^{LAM}=\frac{3!}{4!}\Gamma_{ijkl}\left(0,0\right)$$

(11) $$\delta_{ijkl}^{HEX}=\frac{18}{4!{\left(\sqrt{3}\right)}^{4}}\left[\Gamma_{ijkl}\left(0,0\right)+4\Gamma_{ijkl}\left(0,1\right)\right]$$

(12) $$\delta_{ijkl}^{BCC}=\frac{36}{4!{\left(\sqrt{6}\right)}^{4}}\left[\Gamma_{ijkl}\left(0,0\right)+8\Gamma_{ijkl}\left(0,1\right)+2\Gamma_{ijkl}\left(0,2\right)+4\Gamma_{ijkl}\left(1,2\right)\right]$$

The set of numbers $\left(h_{1},h_{2}\right)$ in Equations (10)–(12) indicates the relative angles between the four scattering vectors ${\vec{k}}_{1}$, ${\vec{k}}_{2}$, ${\vec{k}}_{3}$, and ${\vec{k}}_{4}$. We define $h_{1}$ and $h_{2}$ as ${\left|{\vec{k}}_{1}+{\vec{k}}_{2}\right|}^{2}\equiv h_{1}\cdot {\left(k*\right)}^{2}$ and ${\left|{\vec{k}}_{1}+{\vec{k}}_{4}\right|}^{2}\equiv h_{2}\cdot {\left(k*\right)}^{2}$, respectively. Then, it can be shown that ${\left|{\vec{k}}_{1}+{\vec{k}}_{3}\right|}^{2}=\left(4-h_{1}-h_{2}\right)\cdot {\left(k*\right)}^{2}$. The Landau free energy is to be minimized with respect to $\varsigma_{A}^{}$ and $\varsigma_{B}^{}$ to determine the equilibrium mesophase at a given set of composition, temperature, and pressure.

#### 2.3.2. Method II: Transformation of Order Parameters

Now, let us express our Landau free energy in a more familiar form through the transformation of the order parameters [45,46]. The new order parameters are denoted as $\psi_{1}\left(\vec{r}\right)$ and $\psi_{2}\left(\vec{r}\right)$, which are defined by the following matrix equation:

(13) $$\left[\begin{array}{c} \psi_{1}\\ \psi_{2} \end{array}\right]=\left[\begin{array}{cc} \left(1-\phi_{A}\right)/\eta & -\phi_{A}/\eta \\ 1 & 1 \end{array}\right]\left[\begin{array}{c} \Delta \eta_{A}\\ \Delta \eta_{P} \end{array}\right]=\left[M_{ij}\right]\left[\begin{array}{c} \Delta \eta_{A}\\ \Delta \eta_{P} \end{array}\right]$$

Using this equation, $\psi_{1}\left(\vec{r}\right)$ is given as $\psi_{1}=\left(\Delta \eta_{A}-\Delta \eta_{P}\right)/2\eta$ at $\phi_{A}=1/2$. Thus, the profiles of phase segregating A and B blocks are joined to yield a composite profile in phase with A block. The other order parameter $\psi_{2}\left(\vec{r}\right)$ is determined to be $\psi_{2}=\Delta \eta_{A}+\Delta \eta_{P}=-\Delta \eta_{f}$, which implies the negative fluctuations in free volume fraction. Upon this transformation, the new vertex functions $\bar{\Gamma }$s are obtained from the original vertex functions Γs as

(14) $${\bar{\Gamma }}_{i_{1}\ldots i_{n}}^{\left(n\right)}\left({\vec{k}}_{1},\ldots ,{\vec{k}}_{n}\right)\psi_{i_{1}}\left({\vec{k}}_{1}\right)\cdots \psi_{i_{n}}\left({\vec{k}}_{n}\right)=\Gamma_{i_{1}\ldots i_{n}}^{\left(n\right)}\left({\vec{k}}_{1},\ldots ,{\vec{k}}_{n}\right)\Delta \eta_{i_{1}}\left({\vec{k}}_{1}\right)\cdots \Delta \eta_{i_{n}}\left({\vec{k}}_{n}\right)$$

Then, $\bar{\Gamma }$s are equated to

(15) $${\bar{\Gamma }}_{j_{1}\ldots j_{n}}^{\left(n\right)}=\Gamma_{i_{1}\ldots i_{n}}^{\left(n\right)}M_{i_{1}j_{1}}^{-1}\cdots M_{i_{n}j_{n}}^{-1}$$

where Einstein’s summation convention is used for this tensorial equation.

We will consider nanoscale mesophases, whose structures are defined by characteristic scattering vectors ${\vec{k}}_{1}\in \left\{\pm {\vec{K}}_{n}\right\}$. Regular geometric morphologies are represented by the order parameter $\psi_{1}$ that is treated as a plane wave as $\psi_{1}(\pm {\vec{K}}_{k})=\left(1/\sqrt{n}\right)\zeta_{1}e^{\pm i\varphi_{k}\left(1\right)}$. The remaining $\psi_{2}$ is separated into two parts as $-\psi_{2}=\Delta \eta_{f}$$=-\psi_{2c}-\psi_{2i}$, where the former indicates the excess free volume in phase with the more compressible constituent and the latter represents the excess free volume at the interfaces between domains. While $\psi_{2c}$ is parametrized as $\psi_{2c}(\pm {\vec{K}}_{k})=$$\left(1/\sqrt{n}\right)\zeta_{2c}e^{\pm i\varphi_{k}\left(2c\right)}$, $\psi_{2i}$ should have 1/2 period to locate the interfaces as $\psi_{2i}(\pm 2{\vec{K}}_{k})=$$\left(1/\sqrt{n}\right)\zeta_{2i}e^{\pm i\varphi_{k}\left(2i\right)}$.

Taking the proper mathematical procedure given in the Appendix B, this alternative Landau free energy is formulated as

(16) $$\beta \Delta A={\bar{\Gamma }}_{11}^{}\zeta_{1}^{2}+2{\bar{\Gamma }}_{12}^{}\zeta_{1}^{}\zeta_{2c}^{}+{\bar{\Gamma }}_{22}^{}\zeta_{2c}^{2}+{\bar{\Gamma }}_{22}^{}\left(2k*\right)\zeta_{2i}^{2}-\left|a_{n}\zeta_{1}^{3}+b_{1}\zeta_{1}^{2}\zeta_{2c}\right|+c_{4}\zeta_{1}^{2}\zeta_{2i}+d_{n}\zeta_{1}^{4}$$

where the coefficient $a_{n}$ is given respectively for LAM, HEX, and BCC by

(17) $$a_{n}^{LAM}=0;\;a_{n}^{HEX}=12/\left(3!3^{3/2}\right)\cdot {\bar{\Gamma }}_{111}\left(1\right);\;a_{n}^{BCC}=48/\left(3!6^{3/2}\right)\cdot {\bar{\Gamma }}_{111}\left(1\right)$$

The coefficient $b_{1}$ is given respectively by

(18) $$b_{1}^{LAM}=0;\;b_{1}^{HEX}=12/\left(3!3^{3/2}\right)\cdot \left(3{\bar{\Gamma }}_{112}\left(1\right)\right);\;b_{1}^{BCC}=48/\left(3!6^{3/2}\right)\cdot \left(3{\bar{\Gamma }}_{112}\left(1\right)\right)$$

for LAM, HEX, and BCC. The coefficient $c_{4}$ respectively becomes

(19) $$c_{4}^{LAM}=2/3!\cdot \left(3{\bar{\Gamma }}_{112}\left(4\right)\right);\;c_{4}^{HEX}=6/\left(3!3^{3/2}\right)\cdot \left(3{\bar{\Gamma }}_{112}\left(4\right)\right);\;c_{4}^{BCC}=12/\left(3!6^{3/2}\right)\cdot \left(3{\bar{\Gamma }}_{112}\left(4\right)\right)$$

The quartic coefficient $d_{n}^{}$ is given as

(20) $$d_{n}^{LAM}=\frac{3!}{4!}{\bar{\Gamma }}_{1111}\left(0,0\right)$$

(21) $$d_{n}^{HEX}=\frac{18}{4!{\left(\sqrt{3}\right)}^{4}}\left[{\bar{\Gamma }}_{1111}\left(0,0\right)+4{\bar{\Gamma }}_{1111}\left(0,1\right)\right]$$

(22) $$d_{n}^{BCC}=\frac{36}{4!{\left(\sqrt{6}\right)}^{4}}\left[{\bar{\Gamma }}_{1111}\left(0,0\right)+8{\bar{\Gamma }}_{1111}\left(0,1\right)+2{\bar{\Gamma }}_{1111}\left(0,2\right)+4{\bar{\Gamma }}_{1111}\left(1,2\right)\right]$$

for LAM, HEX, and BCC, respectively.

Differentiating Equation (16) with respect to $\zeta_{2c}^{}$ and $\zeta_{2i}^{}$, and then nullifying those derivatives yield the following conditions:

(23) $$\zeta_{2c}^{}=-\frac{{\bar{\Gamma }}_{12}^{}}{{\bar{\Gamma }}_{22}^{}}\zeta_{1}^{}\pm \frac{b_{1}}{2{\bar{\Gamma }}_{22}^{}}\zeta_{1}^{2};\;\zeta_{2i}^{}=-\frac{c_{4}}{2{\bar{\Gamma }}_{22}^{}\left(2k*\right)}\zeta_{1}^{2}$$

where + and − signs are assigned to $a_{n}\zeta_{1}^{}+b_{1}\zeta_{2c}>0$ and $a_{n}\zeta_{1}^{}+b_{1}\zeta_{2c}<0$, respectively. Replacing $\zeta_{2c}^{}$ and $\zeta_{2i}^{}$ with Equation (23), the free energy becomes in general

(24) $$\beta \Delta A=\left({\bar{\Gamma }}_{11}^{}-\frac{{\bar{\Gamma }}_{12}^{2}}{{\bar{\Gamma }}_{22}^{}}\right)\zeta_{1}^{2}-\left|a_{n}-\frac{b_{1}{\bar{\Gamma }}_{12}^{}}{{\bar{\Gamma }}_{22}^{}}\right|\zeta_{1}^{3}+\left(d_{n}-\frac{b_{1}^{2}}{4{\bar{\Gamma }}_{22}^{}}-\frac{c_{4}^{2}}{4{\bar{\Gamma }}_{22}^{}\left(2k*\right)}\right)\zeta_{1}^{4}\approx \left({\bar{\Gamma }}_{11}^{}-\frac{{\bar{\Gamma }}_{12}^{2}}{{\bar{\Gamma }}_{22}^{}}\right)\zeta_{1}^{2}-\left|a_{n}-\frac{b_{1}{\bar{\Gamma }}_{12}^{}}{{\bar{\Gamma }}_{22}^{}}\right|\zeta_{1}^{3}+d_{n}\zeta_{1}^{4}$$

Equation (20) is our final suggestion of the alternative Landau free energy to find the equilibrated ordered state as its minimum. It can be seen that the effective cubic and quartic coefficients of the free energy contain not only the Gaussian correlation functions but also interaction-dependent ${\bar{\Gamma }}_{ij}^{}$. The vertex coefficient ${\bar{\Gamma }}_{22}^{}$ implies the bulk modulus of the copolymer melt [42]. Thus, the effective quartic coefficient in Equation (24) is further approximated to simply $d_{n}$.

### 2.4. Spinodals and Effective Flory χ

The quadratic form $A_{2}$ of the Landau free energy in Equation (9) can be expressed in the matrix form as

(25) $$\beta A_{2}=\Gamma_{AA}^{}\varsigma_{A}^{2}-2\Gamma_{AB}^{}\varsigma_{A}^{}\varsigma_{B}^{}+\Gamma_{BB}^{}\varsigma_{B}^{2}=\left[\begin{array}{cc} \varsigma_{A}^{} & \varsigma_{B}^{} \end{array}\right]\left[\begin{array}{cc} \Gamma_{AA}^{} & -\Gamma_{AB}^{}\\ -\Gamma_{AB}^{} & \Gamma_{BB}^{} \end{array}\right]\left[\begin{array}{c} \varsigma_{A}^{}\\ \varsigma_{B}^{} \end{array}\right]$$

The phase stability requires the positive definiteness of $A_{2}$. The spinodals are then defined as the border line of stability to require $\det\left[\Gamma_{ij}^{}\right]=0$ at $k^{*}$ or

(26) $$\Gamma_{AA}^{}/\Gamma_{AB}^{}=\Gamma_{AB}^{}/\Gamma_{BB}^{}$$

The same situation occurs in our alternative Landau free energy in Equation (24), where the spinodals are determined by

(27) $${\bar{\Gamma }}_{11}^{}-{\bar{\Gamma }}_{12}^{2}/{\bar{\Gamma }}_{22}^{}=\det\left[{\bar{\Gamma }}_{ij}^{}\right]/{\bar{\Gamma }}_{22}^{}=0$$

These two different equations for spinodals are simply equivalent because $\det\left[{\bar{\Gamma }}_{ij}^{}\right]=\eta^{2}\det\left[\Gamma_{ij}^{}\right]$.

The essence of the phase behavior of diblock copolymer melts is concentrated on effective Flory *χ* parameter. In our previous works [42,46], *χ* was properly elicited from the spinodals to consist of two contributions as $\chi =\chi_{H}+\chi_{S}$. The former $\chi_{H}$ of our *χ* indicates the conventional enthalpic contribution gotten from ${\bar{\Gamma }}_{11}^{}$ in the following way. There are Gaussian and non-Gaussian parts in ${\bar{\Gamma }}_{11}^{}/\eta$ as

(28) $${\bar{\Gamma }}_{11}^{}/\eta =\eta \left(\Gamma_{AA}^{}-2\Gamma_{AB}^{}+\Gamma_{BB}^{}\right)=\eta \left(S_{AA}^{0-1}-2S_{AB}^{0-1}+S_{BB}^{0-1}\right)+\eta \beta v*\left(D_{AA}a_{ni}-2D_{AB}a_{ni}+D_{BB}a_{ni}\right)$$

where the latter non-Gaussian ones give $\chi_{H}$ as

(29) $$\chi_{H}=-\frac{1}{2}\beta v*\left(D_{AA}a_{ni}-2D_{AC}a_{ni}+D_{CC}a_{ni}\right)\eta =\beta \cdot \frac{1}{2}\Delta \bar{\varepsilon }\cdot \left|u\left(\eta \right)\right|$$

The symbol $\Delta \bar{\varepsilon }$ (=${\bar{\varepsilon }}_{AA}+{\bar{\varepsilon }}_{BB}-2{\bar{\varepsilon }}_{AB}$) implies the exchange energy between ${\bar{\varepsilon }}_{ij}$’s. Unlike incompressible situations, $\chi_{H}$ possesses density dependence because of u(η). Meanwhile, ${\bar{\Gamma }}_{12}$ (=$\eta /2\cdot \left(\Gamma_{AA}^{}-\Gamma_{BB}^{}\right)$) is analyzed to be

(30) $${\bar{\Gamma }}_{12}=\frac{\eta }{2}\beta v*\left(D_{AA}a_{ni}-D_{BB}a_{ni}\right)=\frac{1}{2}\beta \left({\bar{\varepsilon }}_{AA}-{\bar{\varepsilon }}_{BB}\right)\cdot \eta \frac{du}{d\eta }$$

where ${\bar{\varepsilon }}_{AA}-{\bar{\varepsilon }}_{BB}$ indicates disparity in self dispersion interactions between constituent blocks. The remaining vertex function ${\bar{\Gamma }}_{22}$ (=$\sum \Gamma_{ij}^{}/4$) is the average of $\Gamma_{ij}$. It was shown in our previous works [42,46] that ${\bar{\Gamma }}_{22}\approx B_{T}/\eta^{2}$, where $B_{T}$ ($\equiv \eta {\left.\partial P/\partial \eta \right)}_{T}$) is the bulk modulus of the copolymer. Therefore, ${\bar{\Gamma }}_{12}^{2}/{\bar{\Gamma }}_{22}^{}$$\propto {\left[{\bar{\varepsilon }}_{AA}-{\bar{\varepsilon }}_{CC}\right]}^{2}/B_{T}$ dominantly. As ${\bar{\Gamma }}_{12}^{2}/{\bar{\Gamma }}_{22}^{}$ is always positive, it hampers phase stability. The latter $\chi_{S}$ of our *χ* represents the entropic contribution to phase stability as

(31) $$\chi_{S}=\frac{1}{2\eta }\cdot \frac{{\bar{\Gamma }}_{12}^{2}}{{\bar{\Gamma }}_{22}^{}}$$

which is associated with volume fluctuations [42,46]. In general, a component with larger ${\bar{\varepsilon }}_{jj}$ has a stronger cohesive energy and thus smaller compressibility (larger $\eta_{\phi_{j}\to 1}$) than the other. Therefore, $\chi_{S}$ vanishes for the copolymers with the same ${\bar{\varepsilon }}_{jj}$s or compressibility. The determinant $\det\left[\Gamma_{ij}^{}\right]$ can then be re-written as

(32) $$\det\left[\Gamma_{ij}^{}\right]=\frac{1}{\eta^{2}}\det\left[{\bar{\Gamma }}_{ij}^{}\right]=\frac{{\bar{\Gamma }}_{22}^{}}{\eta }\left\{\eta \left(S_{AA}^{0-1}-2S_{AB}^{0-1}+S_{BB}^{0-1}\right)-2\chi \right\}$$

This *χ* is capable of predicting all types of block copolymer phase behaviors.

In response to pressure, $\chi_{H}$ and $\chi_{S}$ behave in the opposite way to each other. Upon pressurization, the increased *η* augments $\chi_{H}$, whereas the increased $B_{T}$ diminishes $\chi_{S}$. In the case that $|{\bar{\varepsilon }}_{AA}-{\bar{\varepsilon }}_{BB}|\to$0, $\chi_{S}/\chi \to$0 and $\chi_{H}$ becomes a dominating contribution to *χ*. Therefore, pressurization leads the system to a deeper segregation, which is the conventional behavior or barotropicity. In the case that $\left|{\bar{\varepsilon }}_{AA}-{\bar{\varepsilon }}_{BB}\right|/{\bar{\varepsilon }}_{AA}$ is more sizable, $\chi_{S}/\chi$ gets more substantial. The applied pressure enhances $B_{T}$, and then $\chi_{S}$ as well as *χ* is suppressed by $B_{T}$, which is the baroplasticity.

## 3. Discussions

### 3.1. Symbolic Arguments on Critical Point

A critical point (CP) or continuous transition point occurs when the spinodal line meets the ODT and OOT lines. The partial minimization of the free energy in Equation (8), with respect to $\varsigma_{B}$ is obtained by $\partial \Delta A/\partial \varsigma_{B}=0$, which yields $\varsigma_{B}=\left(\Gamma_{AB}/\Gamma_{BB}\right)\varsigma_{A}+O\left(\varsigma_{B}^{2}\right)$. When approaching its CP, $\varsigma_{B}\to \left(\Gamma_{AB}/\Gamma_{BB}\right)\varsigma_{A}$ and higher-order terms can be ignored. Putting this $\varsigma_{B}$ back into the free energy yields the following symbolic equation:

(33) $$\beta \Delta A=\tau \varsigma_{A}^{2}+\alpha \varsigma_{A}^{3}+\delta \varsigma_{A}^{4}$$

where $\tau \equiv \Gamma_{AA}^{}-\Gamma_{AB}^{2}/\Gamma_{BB}$ ($\propto \det\left[\Gamma_{ij}^{}\right]$) serves as an effective temperature. The condition that τ>0 indicates the disordered state, above the spinodals for the conventional UODT-type copolymers but below the spinodals for LDOT-type copolymers. The situation that τ<0 implies the ordered state. The remaining effective coefficients α and δ are given by

(34) $$\alpha \equiv -\left|\alpha_{AAA}-3\alpha_{AAB}\frac{\Gamma_{AB}}{\Gamma_{BB}}+3\alpha_{ABB}{\left\{\frac{\Gamma_{AB}}{\Gamma_{BB}}\right\}}^{2}-\alpha_{BBB}{\left\{\frac{\Gamma_{AB}}{\Gamma_{BB}}\right\}}^{3}\right|$$

and

(35) $$\delta \equiv \delta_{AAAA}-4\delta_{AAAB}\frac{\Gamma_{AB}}{\Gamma_{BB}}+2\left\{\delta_{AABB}+\delta_{ABAB}+\delta_{ABBA}\right\}{\left\{\frac{\Gamma_{AB}}{\Gamma_{BB}}\right\}}^{2}-4\delta_{ABBB}{\left\{\frac{\Gamma_{AB}}{\Gamma_{BB}}\right\}}^{3}+\delta_{BBBB}{\left\{\frac{\Gamma_{AB}}{\Gamma_{BB}}\right\}}^{4}$$

where it is perceived that $\delta_{AABB}=\delta_{BBAA}$, $\delta_{ABAB}=\delta_{BABA}$, and $\delta_{ABBA}=\delta_{BAAB}$. In other cases, such as $\delta_{ABBB}$ or $\delta_{AAAB}$, the vertex functions under the permutation of indices are equivalent. It will be seen that *δ* is dominated by $\delta_{AAAA}$ and $\delta_{BBBB}$. A CP is obtainable if the cubic coefficient *α* vanishes. It is clearly seen in Equation (34) that the energetics come into play in finding the CP through $\Gamma_{ij}$s.

In case of using the alternative Landau free energy, the same symbolic expression for the free energy is understood as

(36) $$\beta \Delta A=\bar{\tau }\zeta_{A}^{2}+\bar{\alpha }\zeta_{A}^{3}+\bar{\delta }\zeta_{A}^{4}$$

where the effective coefficients are given as

(37) $$\bar{\tau }={\bar{\Gamma }}_{11}^{}-\frac{{\bar{\Gamma }}_{12}^{2}}{{\bar{\Gamma }}_{22}^{}};\;\bar{\alpha }=-\left|a_{n}-\frac{b_{1}{\bar{\Gamma }}_{12}^{}}{{\bar{\Gamma }}_{22}^{}}\right|;\;\bar{\delta }\approx d_{n}$$

Our alternative Landau free energy in Equation (24) suggests that a CP is obtainable if $a_{n}-b_{1}{\bar{\Gamma }}_{12}^{}/{\bar{\Gamma }}_{22}^{}$ = 0 along with the condition that ${\bar{\Gamma }}_{11}^{}-{\bar{\Gamma }}_{12}^{2}/{\bar{\Gamma }}_{22}^{}=0$ or $\det\left[{\bar{\Gamma }}_{ij}^{}\right]=0$. It is also observed that the energetics play their role in finding the CP due to ${\bar{\Gamma }}_{12}^{}$ and ${\bar{\Gamma }}_{22}^{}$.

The mathematical structure of the effective cubic term in either Equation (34) or Equation (37) demonstrates the existence of CP for an A-b-B copolymer with or without disparity in ${\bar{\varepsilon }}_{jj}$s unlike liquid-solid and nematic-isotropic transitions. The continuous transition for the copolymer with a finite chain size is of course to be destroyed due to concentration fluctuations to that turn to a weak first-order transition [55]. Nonetheless, this mean-field analysis is amenable and neat. It is still of importance because the mean-field behaviors are restored if $N_{c}\to \infty$ [56]. Furthermore, our Landau free energy works as the starting point for any fluctuation correction analyses.

### 3.2. Temperature Dependence of Ordering Transitions

#### 3.2.1. UODT System

In this section, we use the Landau free energy in Equation (8) or Equation (24) to discuss various phase behaviors of molten A-b-B copolymers through numerically determining equilibrium mesophases and their stability. Consider first the phase behaviors of PS-b-PBD, which is quite a typical UODT-type block copolymer. In order to probe its phase behavior, our equation-of-state model requires three homopolymer parameters: the self-interaction parameter ${\bar{\varepsilon }}_{jj}$, monomer diameter $\sigma_{j}$, and chain size $N_{j}$. The sets of homopolymer parameters for PS and PBD are given in Table 1, where a composite parameter $N_{j}\pi \sigma_{j}^{3}/6M_{j}$ carrying the ratio of $N_{j}$ to molecular weight $M_{j}$ is provided. So, $N_{j}$ can be determined from the experimental molecular weight of a polymer or $N_{j}$ is directly given. Cross interactions between different polymers are characterized by ${\bar{\varepsilon }}_{ij}$, which is an adjustable parameter and determined by fitting the phase behaviors of a given block copolymer system or those of the corresponding blends. The ratio ${\bar{\varepsilon }}_{ij}/{\left({\bar{\varepsilon }}_{ii}{\bar{\varepsilon }}_{jj}\right)}^{1/2}$ for PS-b-PBD is determined to be 0.99565 from fitting binodal points of PS/PBD blends [41,57] and also the ordering transitions of PS-b-PBD [41,45,58,59].

The characteristic squared wavenumber $x^{*}$ (= ${\left(R_{G}^{}k^{*}\right)}^{2}$) obtained at the minimum of $\det\left[\Gamma_{ij}\right]$ gives the information on the domain size. In Table S1 of Supplemental Materials (SM), $x^{*}$ for PS-b-PBD is tabulated against $\phi_{A}$. It is seen from this table that $x^{*}$ is symmetric to $\phi_{A}=\phi_{PS}$ for the typical UODT systems such as PS-b-PBD, with little to no disparity in self dispersion interactions ${\bar{\varepsilon }}_{jj}$s.

Prior to the actual phase behaviors, let us briefly take a look at a hypothetical A-b-B diblock copolymer with $N_{c}$ = 400, where each block has the same homopolymer parameters as those of PS and ${\bar{\varepsilon }}_{ij}=0.99565{\left({\bar{\varepsilon }}_{ii}{\bar{\varepsilon }}_{jj}\right)}^{1/2}$. The exchange energy then becomes $\Delta \bar{\varepsilon }/k$ = 35.73 K. In Table S2 of SM, $\Gamma_{ij}^{}$s are tabulated for this copolymer at some selected $\phi_{A}$s. As is seen in this table, $\Gamma_{AA}^{}=\Gamma_{AB}^{}=\Gamma_{BB}^{}$ at $\phi_{A}$ = 0.5. In this case, ${\bar{\Gamma }}_{12}=0$ due to ${\bar{\varepsilon }}_{AA}={\bar{\varepsilon }}_{BB}$. Its phase behavior at ambient pressure is identical to that of the incompressible A-b-B copolymer melt discussed by Leiber. In Table S3 of SM, $\Gamma_{ijk}^{}$s are tabulated against the composition $\phi_{A}$. It is noted that $\Gamma_{iii}^{}$ is negative and large in its magnitude, whereas $\Gamma_{AAB}^{}$ or $\Gamma_{ABB}^{}$ is positive and mostly small. It is shown that $\Gamma_{AAA}^{}=\Gamma_{BBB}^{}$ and $\Gamma_{AAB}^{}=\Gamma_{ABB}^{}$ at $\phi_{A}$ = 0.5. Therefore, α in Equation (34) is nullified at this composition to yield the CP, where $N\chi_{c}$ = 10.49487. In case of PS-b-PBD with $N_{c}$ = 400, there is a small difference in ${\bar{\varepsilon }}_{jj}$s between PS and PBD with $\left|{\bar{\varepsilon }}_{PS}-{\bar{\varepsilon }}_{PBD}\right|/{\bar{\varepsilon }}_{PS}$ = ~0.01. The exchange energy for this copolymer is $\Delta \bar{\varepsilon }/k$ = 35.66 K. Based on $\Gamma_{ij}^{}$s tabulated for PS-b-PBD in Table S4 of SM, $\Gamma_{ij}^{}$s are not identical at $\phi_{A}$ = $\phi_{PS}$ = 0.5. Therefore, α cannot vanish at $\phi_{A}$ = 1/2. The CP of PS-b-PBD is found to be at $\phi_{A}$ = 0.50095 (>1/2) and at $N_{c}\chi$ = 10.49494 because of the small disparity in ${\bar{\varepsilon }}_{jj}$s.

Using the alternative Landau free energy in Equation (24), the CP of PS-b-PBD system turns out to be $\phi_{A}$ = 0.50095, which is identical to the one using Equation (8) at least up to 9 decimal places. These results prove the equivalence of our two different Landau free energies even though the first method does not provide the profile for the free volume fraction. It needs to be mentioned that the threshold or maximum of the spinodals for PS-b-PBD copolymer is located at $\phi_{A}$ = 0.50069, which is slightly moved to the copolymer with more PBD than at the CP. The shift of the CP is more vivid in the next copolymer exhibiting LDOT.

**Figure 1:**
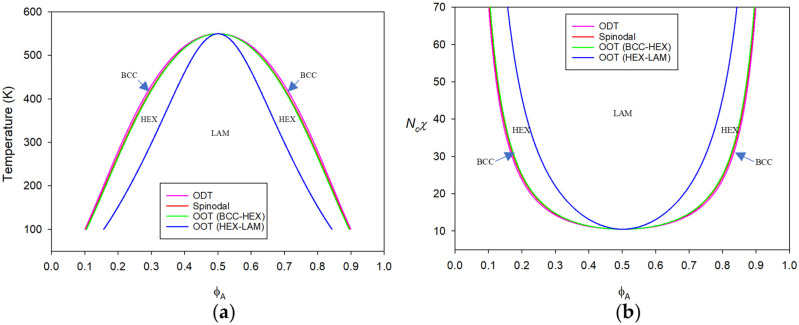
Phase diagram for molten PS-b-PBD with $N_{c}$ = 400 plotted against PS (A) volume fraction $\phi_{A}$ in terms of: (**a**) absolute temperature and (**b**) the relevant parameter $N_{c}\chi$. The disparity in ${\bar{\varepsilon }}_{jj}$s is $\left|{\bar{\varepsilon }}_{PS}-{\bar{\varepsilon }}_{PBD}\right|/{\bar{\varepsilon }}_{PS}$ = 0.010 and the exchange energy is $\Delta \bar{\varepsilon }/k$ = 35.66 K. As this disparity is quite small, the phase boundaries are almost symmetrical with the CP at $\phi_{A}$ = 0.50095. The arrows indicate BCC mesophase in the narrow region between ODT and spinodals.

Starting from the CP of PS-b-PBD copolymer melts, all the transition points at ambient pressure are to be determined by minimizing the Landau free energy given in Equation (8). Using the vertex coefficients as given in Tables S3 to S7 of SM, various transition points are obtained by numerically solving both $\partial \Delta A/\partial \varsigma_{A}=0$ and $\partial \Delta A/\partial \varsigma_{B}=0$. In Figure 1a, the transition temperatures are plotted against $\phi_{A}$. Because of the small $\left|{\bar{\varepsilon }}_{PS}-{\bar{\varepsilon }}_{PBD}\right|$, the phase diagram is almost symmetrical. The phase diagram can also be drawn in terms of the well-known relevant parameter for phase segregation, i.e., $N_{c}\chi$, as shown in Figure 1b. As was mentioned in the previous section, the effective Flory *χ* is a composite function of various molecular parameters as $\chi =\chi_{H}+\chi_{S}$. In Table 2, $N_{c}\chi$ along with $\chi_{H}$ and $\chi_{S}$ for the symmetric PS-b-PBD copolymer with $N_{c}$ = 400 is tabulated at the selected temperatures and at 0.1 MPa. In this system, *χ* is almost equal to $\chi_{H}$. This typical UODT-type copolymer shows the decreasing tendency of *χ* as $\chi_{H}\sim 1/T$, as seen in this table.

In drawing Figure 1, the Landau free energy in Equation (8) is used. If we use the Landau free energy given in Equation (24), where there is only one order parameter amplitude to determine through solving $\partial \Delta A/\partial \zeta_{1}=0$, we get almost the identical phase diagram. The spinodals from the two methods are perfectly identical. The ODT from disorder to BCC is different only by ~0.01 K between the two methods. The calculated differences in OOTs from the two methods are ~0.0007 K and ~0.102 K for BCC-HEX and HEX-LAM OOTs, respectively.

#### 3.2.2. LDOT System

Our second system is a molten diblock copolymer from PS and poly(vinyl methyl ether) (PVME). The corresponding PS/PVME blend is a widely studied blend system that reveals the miscibility between PS and PVME and also the lower critical solution temperature behavior [60]. The origin of their miscibility is considered to be the weak hydrogen bond between the aromatic hydrogen (C-H) and ether oxygen (-O-) [61]. In analyzing copolymer phase behavior, all the molecular parameters for PS-b-PVME are given in Table 1. The cross interaction ${\bar{\varepsilon }}_{ij}$ for this copolymer is determined to be ${\bar{\varepsilon }}_{ij}/{\left({\bar{\varepsilon }}_{ii}{\bar{\varepsilon }}_{jj}\right)}^{1/2}$ = 1.00264 from the binodal points of the corresponding PS/PVME blends, where this ${\bar{\varepsilon }}_{ij}$ yields $\Delta \bar{\varepsilon }/k$ = −6.637 K and the calculated transition temperatures are similar to the experimental values [41,44,60].

The characteristic squared wavenumber $x^{*}$ (= ${\left(R_{G}^{}k^{*}\right)}^{2}$) for PS-b-PVME is tabulated against $\phi_{A}=\phi_{PS}$ in Table S1 of SM. Unlike the typical UODT systems such as PS-b-PBD, it is seen from this table that $x^{*}$ is slightly asymmetric to $\phi_{A}$. The ratio of $x_{\phi_{A}=0.9}^{*}$ to $x_{\phi_{A}=0.1}^{*}$ is shown to be $x_{\phi_{A}=0.9}^{*}/x_{\phi_{A}=0.1}^{*}=1.0011$, which implies that the domain size of the copolymer richer in PS is shrunken compared with that richer in PVME.

**Figure 2:**
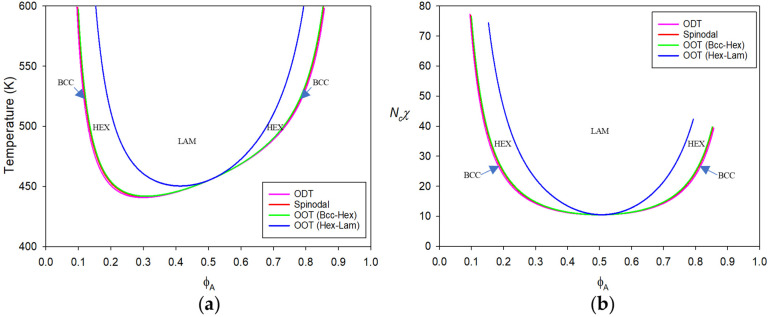
Phase diagram for molten PS-b-PVME with $N_{c}$ = 20,000 plotted against PS (A) volume fraction $\phi_{A}$ in terms of: (**a**) absolute temperature and (**b**) the relevant parameter $N_{c}\chi$. The disparity in ${\bar{\varepsilon }}_{jj}$s is sizable as $\left|{\bar{\varepsilon }}_{PS}-{\bar{\varepsilon }}_{PBD}\right|/{\bar{\varepsilon }}_{PS}$ = 0.113 and the exchange energy is $\Delta \bar{\varepsilon }/k$ = −6.637 K. In this situation, the phase boundaries are skewed towards more compressible PVME side, but with the CP at $\phi_{A}$ = 0.50974. The arrows indicate BCC mesophase in the narrow region between ODT and spinodals.

The effect of disparity in self dispersion interactions appears drastically in PS-b-PVME, which exhibits $\left|{\bar{\varepsilon }}_{PS}-{\bar{\varepsilon }}_{PVME}\right|/{\bar{\varepsilon }}_{PS}$ = 0.113. Since ${\bar{\varepsilon }}_{PS}>{\bar{\varepsilon }}_{PVME}$, PS is denser and less compressible than PVME. PS and PVME are compatible with $\Delta \bar{\varepsilon }<0$ due to the aforementioned weak H-bonds between them. In Table 3, we listed the theoretical χ for PS-b-PVME with $N_{c}$ = 20,000 at $\phi_{A}$ = 1/2 as a function of temperature while fixing pressure to 0.1 MPa. It is seen that the energetic $\chi_{H}\propto$$\Delta \bar{\varepsilon }/T$ is negative and decreases with temperature. However, there is comparable entropic $\chi_{S}$$\propto {\left[{\bar{\varepsilon }}_{AA}-{\bar{\varepsilon }}_{CC}\right]}^{2}/B_{T}$, which is always positive and grows with temperature. As a result of these two competing actions, the copolymer is in the disordered state at lower temperatures but reveals nanoscopic phase separation upon heating or LDOT caused by compressibility difference. The phase separation induced in this way requires a large chain size to suppress the combinatorial entropy. In Figure 2, the spinodal points (red line) are plotted against $\phi_{A}$. It is seen that the spinodal line is seriously asymmetric because of the substantial disparity in ${\bar{\varepsilon }}_{jj}$s. More precisely, the threshold or minimum of the spinodal line is skewed towards more compressible PVME-rich side at $\phi_{A}$ = ~0.305. This phenomenon is caused by the fact that the positivity of $\chi_{S}$ always hampers phase stability, which is stronger in the side rich in more compressible PVME. However, this minimum is not the CP. The calculated CP using the free energy in Equation (8) is found to be $\phi_{A}$ = 0.50974, rich in less compressible component PS. This action is caused by the fact that ${\bar{\varepsilon }}_{PS}>{\bar{\varepsilon }}_{PVME}$. The stronger binding of PS monomers in turn yields that $\Gamma_{AA}^{}<\Gamma_{AB}^{}<\Gamma_{BB}^{}$, as seen in Table S4 of SM. The system rich in denser component has smaller volume. Therefore, at the CP with a continuous transition, the copolymer system strives to search the composition of comparable volumes of the two components. Henceforth, the critical composition should be $\phi_{A}>1/2$ in order to add more volume of less compressible and denser component. This result is in sharp contrast to the phase behavior of the corresponding blend, where the threshold point in the spinodal line is indeed the CP. Using the Landau free energy in Equation (24) yields the CP of PS-b-PVME at $\phi_{A}$ = 0.50974, which is identical to that from Equation (8) up to six decimal places.

Starting from the CP of PS-b-PVME copolymer, all the transition points at ambient pressure are to be determined again using the vertex coefficients as in Tables S2–S7 of SM. Because of the asymmetry in $x^{*}$, the $\delta_{ijkl}$s for the copolymer is minutely different from these given in those tables when approaching both extremes at $\phi_{A}\to 0$ and $\phi_{A}\to 1$. Figure 2a displays all the phase boundaries as well as spinodals for PS-b-PVME in terms of the absolute temperature. The substantial disparity in ${\bar{\varepsilon }}_{jj}$s between PS and PVME forces all those lines to skew, as seen in this figure. In Figure 2b, the phase diagram is redrawn in terms of $N_{c}\chi$, which is quite slanted for molten PS-b-PVME. To get the data in Figure 2, the Landau free energy in Equation (8) is used. Even if the free energy in Equation (24) is used instead, it is observed that we still get almost the identical phase diagram. The spinodals from the two methods are perfectly identical. The ODT from disorder to BCC for the copolymer at $\phi_{A}$ = 0.1 using Equation (8) is different by ~0.2 K from that using Equation (24). The calculated difference in OOTs from BCC to HEX using the two methods for the copolymer at the same composition is found to be ~0.2 K. The predicted HEX-LAM OOTs using Equations (8) and (24) are 1040.074 K and 1056.842 K, respectively. In this case, ΔT reaches 16.8 K. However, the agreement between the two methods is satisfactory considering that the difference is less than 2% even in this unreachable temperature region.

The compressible nature and disparity in ${\bar{\varepsilon }}_{jj}$s for PS-b-PVME gives the difference in the order parameter amplitudes. The ratio $\varsigma_{A}/\varsigma_{B}$ is shown to be ~1.04 for ODT and BCC-HEX OOT for the copolymer at $\phi_{A}$ = 0.1. At other compositions, $\varsigma_{A}/\varsigma_{B}>1$, which reflects the fact that PS is denser than PVME. As the transition temperature is further increased in case of HEX-LAM OOT for the copolymer at the same composition, $\varsigma_{A}/\varsigma_{B}$ is increased to become ~1.11. The density difference between PS and PVME should grow with thermal expansion.

### 3.3. Pressure Dependence of Ordering Transitions

**Figure 3:**
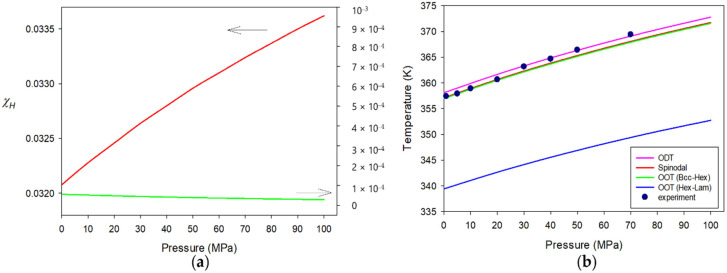
Pressure responses of (**a**) $\chi_{H}$ as well as $\chi_{S}$ at T = 365 K and (**b**) various transitions for molten PS-b-PI with $N_{c}$ = 327.4 (*M_w_* = 16,500) at $\phi_{A}$ = $\phi_{PS}$ = 0.442. The symbols in plot (**b**) indicate the experimental ODT data measured by Hajduk et al. The arrows indicate the proper axes for $\chi_{H}$ and $\chi_{S}$.

In this section, we discuss the responses of diblock copolymers to pressure. The first system to consider is PS-b-PI copolymer, whose ordering transition temperatures have been reported by Hajduk et al. [33,62]. The requisite molecular parameters are also given in Table 1. It is seen in this table that $\left|{\bar{\varepsilon }}_{PS}-{\bar{\varepsilon }}_{PI}\right|/{\bar{\varepsilon }}_{PS}$ = 0.012, which is similar to that for PS-b-PBD. The cross interaction parameter ${\bar{\varepsilon }}_{SI}=$$0.99680{\left({\bar{\varepsilon }}_{PS}{\bar{\varepsilon }}_{PI}\right)}^{1/2}$ is determined from fitting the CP (388 K) of PS/PI blend with molecular weights of 2117 and 2594, reported by Rudolf and Cantow [63], and adjusted by comparison with the ODT data for PS-b-PI with *M_w_* = 8000/8500 [33]. Figure 3a depicts the two contributions to *χ* against pressure for PS-b-PI at $\phi_{A}$ = $\phi_{PS}$ = 0.442 and at T = 365 K. As is now expected from $\left|{\bar{\varepsilon }}_{PS}-{\bar{\varepsilon }}_{PI}\right|$ for this copolymer, Flory *χ* is mostly given by $\chi_{H}$ along with $O(\chi_{S})\sim 10^{-5}$. The enthalpic $\chi_{H}$ increases upon pressurization. Although $\chi_{S}$ goes in a reverse way due to the bulk modulus of the copolymer, the effective Flory *χ* follows $\chi_{H}$ to be strengthened by the applied pressure. In Figure 3b, all the transition points for the copolymer at the same composition are plotted as a function of P. The pressure coefficient, $\Delta T_{trs}/\Delta P$, of the ordering transition is predicted to be ~15 K/100 MPa, which describes well the experimental value of ~17 K/100 MPa for the copolymer with *M_w_* = 16,500 or $N_{c}$ = 327.4 [33]. This type of pressure response is barotropicity, as already mentioned.

**Figure 4:**
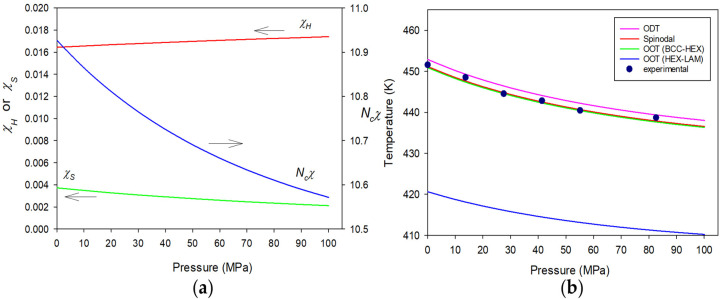
Pressure responses of (**a**) $\chi_{H}$ and $\chi_{S}$ along with $N_{c}\chi$ at 445 K, and (**b**) various transitions for molten PS-b-PEHA with $N_{c}$ = 541.8 (*M_w_* = 23,000) at $\phi_{A}$ = $\phi_{PS}$ = 0.42. The symbols in plot (**b**) indicate the experimental ODT data for the copolymer measured by Lee et al. The arrows indicate the proper axes for $\chi_{H}$, $\chi_{S}$, and $N_{c}\chi$.

Our next system is the copolymer from PS and poly(ethyl hexyl acrylate) (PEHA). The PS-b-PEHA diblock copolymer exhibits a completely reverse response to pressure, as was measured using light scattering (cloud points) and small angle neutron scattering [40]. This copolymer is a member of baroplastic systems, whose nanoscopic phase separation and pressure response were first studied by Mayes and co-workers [38]. Again, all the necessary molecular parameters for PS and PEHA are given in Table 1. It is seen in this table that $\left|{\bar{\varepsilon }}_{PS}-{\bar{\varepsilon }}_{PEHA}\right|/{\bar{\varepsilon }}_{PS}$ = 0.086, which is quite larger than that for PS-b-PBD. The cross-interaction parameter of ${\bar{\varepsilon }}_{S-EHA}=$$0.99880{\left({\bar{\varepsilon }}_{PS}{\bar{\varepsilon }}_{PEHA}\right)}^{1/2}$ is the optimized one to fit the phase behavior of PS-b-PEHA with *M_w_* = 23,000 or $N_{c}$ = 529.581. In Figure 4a, we display *χ* and its two contributions, $\chi_{H}$ and $\chi_{S}$, for the copolymer at $\phi_{A}$ = $\phi_{PS}$ = 0.42 and at T = 445 K. It is observed in this figure that $\chi_{S}$ is near 20% of $\chi_{H}$ at ambient pressure. As pressure is increased, the enthalpic $\chi_{H}$ is increased due to densification. The entropic $\chi_{S}$ is suppressed by the applied pressure to have $\chi_{S}/\chi_{H}$~ 0.12 at P = 100 MPa. As a result, the effective *χ* becomes a decreasing function of pressure. Figure 4b depicts all the transition temperatures for the copolymer at this composition. The decrease of its ODT is predicted to be $\Delta T_{trs}/\Delta P$ = −16 K/100 MPa, which matches well with the scattering result [40].

**Figure 5:**
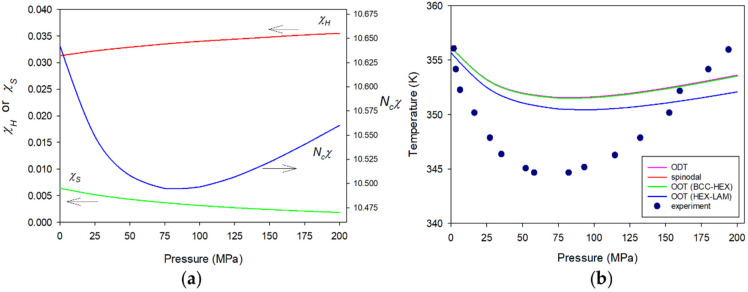
Pressure responses of (**a**) $N_{c}\chi$ along with its two contributions, $\chi_{H}$ and $\chi_{S}$, at 352.5 K, and (**b**) various transitions for molten PEE-b-PDMS with $N_{c}$ = 282.58 (*M_w_* = 10,700) at $\phi_{A}$ = $\phi_{PEE}$ = 0.52. The symbols indicate the experimental ODT data for the copolymer at $\phi_{A}$ = 0.50 measured by Schwahn et al. The arrows indicate the proper axes for $\chi_{H}$, $\chi_{S}$, and $N_{c}\chi$.

Our third system is the diblock copolymer from poly(ethyl ethylene) (PEE) and poly(dimethyl siloxane) (PDMS). The PEE-b-PDMS copolymer is one of UODT-type block copolymers. However, its response to pressure is abnormal in the sense that the copolymer reveals the retreat of its ordering temperatures in the low pressure region and then resurgence of the transition temperatures in the high pressure region [46,64]. This anomalous pressure response of the copolymer can be understood by the subtle balance of $\chi_{H}$ and $\chi_{S}$. In describing the copolymer, the necessary molecular parameters are given in Table 4. The key elements there are $\left|{\bar{\varepsilon }}_{EE}-{\bar{\varepsilon }}_{DMS}\right|/{\bar{\varepsilon }}_{EE}$ = 0.108 and ${\bar{\varepsilon }}_{EE-DMS}$ = $0.99654{\left({\bar{\varepsilon }}_{EE}{\bar{\varepsilon }}_{DMS}\right)}^{1/2}$, determined by fitting the phase behaviors of symmetric PEE-b-PDMS with *M_w_* = ~10,700. Using these parameters, $\Delta \bar{\varepsilon }$ is unfavorable as $\Delta \bar{\varepsilon }/k$ = 27.158 K. Figure 5a depicts the effective *χ* along with its two contributions, $\chi_{H}$ and $\chi_{S}$, plotted against pressure for PEE-b-PDMS at $\phi_{A}$ = $\phi_{PEE}$ = 0.52 and at 352.5 K. At ambient pressure, it is seen that $\chi_{S}$ is near 20% of $\chi_{H}$, and at 100 MPa $\chi_{S}$ drops to near 10% of $\chi_{H}$ just as in the case of PS-b-PEHA. However, unlike PS-b-PEHA, the increase of $\chi_{H}$ is more rapid, so that $\chi_{S}$ becomes just 5% of $\chi_{H}$ at 200 MPa. Therefore, $\chi_{H}$ regains the control of the phase behaviors. The transition temperatures of this copolymer are shown in Figure 5b against pressure from 0.1 to 200 MPa. In this figure, the baroplastic, followed by barotropic responses of PEE-b-PDMS, are clearly demonstrated in agreement with the experiment.

We have revisited the Landau free energy for A-b-B diblock copolymer melts with diverse types of phase behaviors from the viewpoint of their response to temperature or pressure. Being analytical with one harmonic for order parameters, the present work deals with the classical nanostructures. For the equilibration of other mesophases, it is necessary to use our self-consistent field theory for the copolymers, which was developed a few years ago [47,48,49]. All of our works are based on the restricted chain model with the identical monomer diameters $\sigma_{j}$s. This restriction can be alleviated to allow for the variation of $\sigma_{j}$s. The present mean-field Landau energy with its correct cubic term exhibits continuous transitions or CPs, whose locations are dependent on disparity in ${\bar{\varepsilon }}_{jj}$s. When the concentration fluctuations are involved, such mean-field CPs are known to be destroyed to yield the weak first-order transition [55]. The necessary fluctuation correction in one-loop order was suggested by Fredrickson and Helfand [56] for the corresponding incompressible A-b-B diblock copolymer systems utilizing Brazovskii’s Hamiltonian form. One of the present authors also introduced the similar approach for the copolymer melt in case of small $\left|{\bar{\varepsilon }}_{AA}-{\bar{\varepsilon }}_{BB}\right|$ [65]. In Appendix C, we provide the fluctuation correction analysis starting from our Landau free energy in Equation (24).

## 4. Conclusions

Nanoscopic phase behaviors of molten A-b-B diblock copolymers within general disparity in self dispersion interactions are revisited through Landau approach. A continuum space molecular equation of state is first considered to describe such copolymers in the bulk state. A free energy functional is obtained for the inhomogeneous copolymer as that for the corresponding Gaussian chains under the influence of effective two-body interactions from the localized excess equation of state. The free energy functional in the weak segregation regime is then expanded directly as a series in powers of two order parameters, which are fluctuations in A and B block densities ($\langle \Delta \eta_{A}\rangle$ and $\langle \Delta \eta_{B}\rangle$), up to 4th order. The order parameters are treated as the sum of plane waves with their amplitude and phase angles. The Fourier-transformed momentum integral for the Landau free energy is approximated to the finite sum of terms at the scattering vectors $\vec{k}$, whose lengths are the characteristic wavenumber $k^{*}$ signifying the domain sizes of ordered mesophases. A completely alternative Landau free energy is obtained after the transformation of the order parameters to fluctuations in block density difference ($\sim \langle \Delta \eta_{A}-\Delta \eta_{B}\rangle /2\eta$) and negative free volume fraction ($-\langle \Delta \eta_{f}\rangle$). It is shown that those two different Landau free energies are equivalent to yield almost identical ordering transition temperatures.

The analysis of spinodals from the quadratic term of the Landau energy, which are perfectly identical for the direct and alternative ones, leads to effective Flory *χ* as the sum of the conventional enthalpic $\chi_{H}$ for exchange energy ($\propto \Delta \bar{\varepsilon }\left|u\left(\eta \right)\right|$) and entropic $\chi_{S}$ representing disparity in self dispersion interactions mediated by copolymer bulk modulus ($\propto {\left[{\bar{\varepsilon }}_{AA}-{\bar{\varepsilon }}_{CC}\right]}^{2}/B_{T}$). The cubic term of the Landau free energy is shown to be balanced with all the Gaussian cubic vertex coefficients $\Gamma_{ijk}$ in corporation with $\Gamma_{ij}$ to yield its critical point (CP) depending on asymmetry in self dispersion strengths. The quartic terms of the Landau free energy are given mainly by the Gaussian quartic vertex coefficients $\Gamma_{iiii}$s at the proper combinations of the scattering vectors pertinent to the given mesophases.

Taking PS-b-PBD and PS-b-PVME as model systems, the responses of the copolymers to temperature are first discussed. The former copolymer exhibits typical ordering transition upon cooling (UODT), whereas the latter copolymer reveals the reverse ordering transition upon heating (LDOT). The phase boundaries for these copolymers are fully determined by numerically minimizing the Landau energy. The PS-b-PBD copolymer with quite close self-dispersion interactions for both blocks gives a symmetric phase diagram. This phenomenon can be understood because $\chi \approx \chi_{H}$ and $\chi_{S}<<1$, in this case. The copolymer phase behaviors at ambient pressure are almost identical to those of the incompressible A-b-B copolymer. In contrast, PS-b-PVME copolymer possesses quite sizable disparity in self dispersion interactions. The substantially large $\chi_{S}$ in this copolymer develops phase segregation tendency upon heating because of the diminished bulk modulus. The phase boundaries are skewed towards the side rich in more compressible PVME. While PS-b-PBD possesses its CP near the symmetric composition, PS-b-PVME pushes its CP towards the copolymer rich in denser component to match domain volumes to fulfill a continuous transition.

The responses of A-b-B copolymers to pressure are investigated by taking PS-b-PI, PS-b-PEHA, and PEE-b-PDMS as model systems. The PS-b-PI copolymer is barotropic, which is typical for many block copolymers. In this case, *χ* is dominated by $\chi_{H}$, which is augmented by pressurization due to the increased density. On the contrary, the PS-b-PEHA copolymer is baroplastic with more sizable $\chi_{S}$. Pressurization suppresses $\chi_{S}$ because of the copolymer bulk modulus, and the decrease in $\chi_{S}$ affects the total *χ* more than the increase in $\chi_{H}$. Anomaly is observed for the PEE-b-PDMS copolymer, because it is baroplastic at lower pressure region and then barotropic in higher pressure region. The reason for this complicated pressure response is because there is a subtle competition between $\chi_{H}$ and $\chi_{S}$, which prevails between the two switches at different stages of pressurization.

## Figures and Tables

**Table 1:** Molecular Parameters for PS and other polymers that form A-b-B copolymers.

Parameters	PS	PBD	PVME	PI	PEHA
$\sigma_{i}$ (Å)	4.039	4.039	3.900 *^a^*	4.350 *^a^*	3.840 *^a^*
${\bar{\varepsilon }}_{ii}/k$ (K)	4107.0	4065.9	3644.8	4057.7	3755.7
$N_{i}\pi \sigma_{i}^{3}/6M_{i}$ (cm^3^/g) *^b^*	0.41857	0.49395	0.42906	0.50209	0.48564
${\bar{\varepsilon }}_{ij}^{}/{\left({\bar{\varepsilon }}_{PS}{\bar{\varepsilon }}_{jj}\right)}^{1/2}$	-	0.99565	1.00264	0.99680	0.99880

*^a^* This discrepancy in monomer diameters is resolved by adopting the conventional Lorentz mixing rule as $\sigma =\left(\sigma_{i}+\sigma_{j}\right)/2$. *^b^* This composite parameter gives the ratio of the chain size $N_{i}$ to molecular weight $M_{i}$.

**Table 2:** The relevant parameter $N_{c}\chi$ and its two contributions, $\chi_{H}$ and $\chi_{S}$, evaluated at selected temperatures for symmetrical PS-b-PBD with $N_{c}$ = 400 *^a^*.

T (K)	*χ_H_*	*χ_S_*	*N_c_χ*
350	0.04573	4.40866 × 10^−5^	18.31053
400	0.03905	4.57456 × 10^−5^	15.63757
450	0.03382	4.71030 × 10^−5^	13.54714
500	0.02962	4.82407 × 10^−5^	11.86625
550	0.02616	4.92196 × 10^−5^	10.48438
600	0.02327	5.00868 × 10^−5^	9.32755
650	0.02081	5.08801 × 10^−5^	8.34431

*^a^* Pressure is fixed to 0.1 MPa.

**Table 3:** The relevant parameter $N_{c}\chi$ and its two contributions, $\chi_{H}$ and $\chi_{S}$, evaluated at selected temperatures for symmetrical PS-b-PVME with $N_{c}$ = 20,000 *^a^*.

T (K)	*χ_H_*	*χ_S_*	*N_c_χ*
425	−0.00668	0.00656	−2.46405
450	−0.00622	0.00664	8.46668
475	−0.00581	0.00672	18.30529
500	−0.00544	0.00680	27.21505
525	−0.00510	0.00687	35.32983
550	−0.00479	0.00693	42.76065

*^a^* Pressure is fixed to 0.1 MPa.

**Table 4:** Molecular parameters for PEE-b-PDMS systems.

Parameters	PEE	PDMS
$\sigma_{i}$ (Å)	3.590 *^a^*	3.952 *^a^*
${\bar{\varepsilon }}_{ii}/k$ (K)	2819.60	2514.50
$N_{i}\pi \sigma_{i}^{3}/6M_{i}$ (cm^3^/g)	0.47823	0.41778
${\bar{\varepsilon }}_{AB}/{\left({\bar{\varepsilon }}_{AA}{\bar{\varepsilon }}_{BB}\right)}^{1/2}$	0.99654	

*^a^* This discrepancy in monomer diameters is resolved by adopting the conventional Lorentz mixing rule as $\sigma =\left(\sigma_{i}+\sigma_{j}\right)/2$.

## Data Availability

The data presented in this study are available on request from the corresponding author.

## Supplementary Materials

The following supporting information can be downloaded at: https://www.mdpi.com/article/10.3390/polym15010030/s1, Table S1: Characteristic squared wavenumber x* at spinodals for PS-b-PBD and PS-b-PVME melts listed at selected compositions $\phi_{A}$s; Table S2: Quadratic vertex coefficients $\Gamma_{ij}$s for A-b-B with ${\bar{\varepsilon }}_{AA}={\bar{\varepsilon }}_{BB}$ at the indicated compositions $\phi_{A}$s; Table S3: Cubic vertex coefficients $\eta^{2}N_{c}\Gamma_{ijk}$s for A-b-B at the indicated compositions $\phi_{A}$s; Table S4: Quadratic vertex coefficients $\Gamma_{ij}$s for PS-b-PBD and PS-b-PVME at the indicated compositions $\phi_{A}$s; Table S5: Quartic vertex coefficients $\eta^{3}N_{c}\delta_{ijkl}^{BCC}/c_{BCC}$s in Equation (12) for BCC-forming PS-b-PBD at the indicated $\phi_{A}$s; Table S6: Quartic vertex coefficients $\eta^{3}N_{c}\delta_{ijkl}^{HEX}/c_{HEX}$s in Equation (11) for cylinder-forming PS-b-PBD at the indicated $\phi_{A}$s; Table S7: Quartic vertex coefficients $\eta^{3}N_{c}\delta_{ijkl}^{LAM}/c_{LAM}$s in Equation (10) for lamella-forming PS-b-PBD at the indicated $\phi_{A}$s.

## Appendix A. Landau Free Energy in a Direct Way

In this appendix, we provide the detailed procedure in deriving the Landau free energy given in Equation (8). The general integral form of the free energy in Equation (7) is approximated to the finite sum of integrands at *n* characteristic scattering vectors ${\vec{K}}_{j}$s signifying a given structure, where $\left|{\vec{K}}_{j}\right|$ is equal to its characteristic structural wavenumber $k^{*}$. The quadratic form $A_{2}$ of the free energy functional in Equation (7) is then given by

(A1) $$\beta A_{2}=\frac{1}{2}\int d{\vec{k}}_{1}\Gamma_{ij}\left({\vec{k}}_{1},-{\vec{k}}_{1}\right)\Delta \eta_{i}({\vec{k}}_{1})\Delta \eta_{j}(-{\vec{k}}_{1})\approx \frac{1}{2}\sum_{{\vec{k}}_{1}\in \left\{\pm {\vec{K}}_{n}\right\}}\Gamma_{ij}^{}\left({\vec{k}}_{1},-{\vec{k}}_{1}\right)\Delta \eta_{i}({\vec{k}}_{1})\Delta \eta_{j}(-{\vec{k}}_{1})$$

There are only 2n such cases. Each $\Delta \eta_{i}(\pm {\vec{k}}_{1})$ is treated as a plane wave with its amplitude $\left(1/\sqrt{n}\right)\varsigma_{j}$ and phase angle $\pm \varphi \left(i\right)$ as $\Delta \eta_{i}(\pm {\vec{k}}_{1})=\left(1/\sqrt{n}\right)\varsigma_{i}e^{\pm i\varphi \left(i\right)}$. Then, Equation (A1) turns to

(A2) $$\beta A_{2}=\Gamma_{AA}^{}\left({\vec{k}}_{1},-{\vec{k}}_{1}\right)\varsigma_{A}^{2}+2\Gamma_{AB}^{}\left({\vec{k}}_{1},-{\vec{k}}_{1}\right)\varsigma_{A}\varsigma_{B}e^{i\left[\varphi \left(A\right)-\varphi \left(B\right)\right]}+\Gamma_{BB}^{}\left({\vec{k}}_{1},-{\vec{k}}_{1}\right)\varsigma_{B}^{2}$$

It is found that all the $\Gamma_{ij}^{}$s are positive, as seen in Tables S2 and S3 of SM. The minimization of Equation (A2) gives

(A3) $$\beta A_{2}=\left[\Gamma_{AA}^{}\left(k^{*}\right)\right.\varsigma_{A}^{2}-2\Gamma_{AB}^{}\left(k^{*}\right)\varsigma_{A}^{}\varsigma_{B}^{}+\left.\Gamma_{BB}^{}\left(k^{*}\right)\varsigma_{B}^{2}\right]$$

where the phase angles need to satisfy $\varphi \left(A\right)-\varphi \left(B\right)=\pi$.

Let us discuss how to obtain higher-order terms in Equation (8) of the main text. This is where the present work takes a step forward with the deepened conception from the previous study [44]. The cubic form $A_{3}$ of the free energy functional has the vertex functions of all possible combinations of indices *i*, *j*, and *k*. The same parametrization of $\Delta \eta_{i}({\vec{k}}_{1})$ and approximating $A_{3}$ to the finite sum of integrands give

(A4) $$\beta A_{3}=\frac{1}{3!}\sum_{{\vec{k}}_{i}\in \left\{\pm {\vec{K}}_{n}\right\}}\Gamma_{ijk}\left({\vec{k}}_{1},{\vec{k}}_{2},{\vec{k}}_{3}\right)\frac{\varsigma_{i}\varsigma_{j}\varsigma_{k}}{{\left(\sqrt{n}\right)}^{3}}e^{i\left(\varphi_{a}\left(i\right)+\varphi_{b}\left(j\right)+\varphi_{c}\left(k\right)\right)}$$

One should bear in mind that the scattering vectors ${\vec{k}}_{1}$, ${\vec{k}}_{2}$, and ${\vec{k}}_{3}$ satisfy ${\vec{k}}_{1}+{\vec{k}}_{2}+{\vec{k}}_{3}=0$ not to nullify $\Gamma_{ijk}\left(h\right)$. These vectors should then form a right triangle to have *h* = 1. The given vertex functions change their signs according to indices. However, the sum of phase angles cannot be decided by their signs, because the sums are not fully independent. All those sums can be determined relative to $\sum \varphi_{m}\left(A\right)\equiv \varphi_{a}\left(A\right)+\varphi_{b}\left(A\right)+\varphi_{c}\left(A\right)$ as follows:

(A5) $$\varphi_{a}\left(i\right)+\varphi_{b}\left(j\right)+\varphi_{c}\left(k\right)=\varphi_{a}\left(A\right)+\varphi_{b}\left(A\right)+\varphi_{c}\left(A\right)-\pi \cdot c_{B}\left(ijk\right)=\sum \varphi_{m}\left(A\right)-\pi \cdot c_{B}\left(ijk\right)$$

where $c_{B}\left(ijk\right)$ is the number of *B* among indices *i*, *j*, and *k*. Inserting Equation (A5) into A4 yields

(A6) $$\beta A_{3}=\frac{1}{3!}\sum_{{\vec{k}}_{i}\in \left\{\pm {\vec{K}}_{n}\right\}}e^{i\left(\varphi_{a}\left(A\right)+\varphi_{b}\left(A\right)+\varphi_{c}\left(A\right)\right)}\cdot \Gamma_{ijk}\left(1\right)\frac{\varsigma_{i}\varsigma_{j}\varsigma_{k}}{{\left(\sqrt{n}\right)}^{3}}e^{-i\cdot \pi c_{B}\left(ijk\right)}$$

where $\Gamma_{ijk}\left(h\right)$ is evaluated at h=1. The minimization of Equation (A6) leads in general to

(A7) $$\beta A_{3}=-\left|\alpha_{AAA}\varsigma_{A}^{3}-3\alpha_{AAB}\varsigma_{A}^{2}\varsigma_{B}^{}+3\alpha_{ABB}\varsigma_{A}^{}\varsigma_{B}^{2}-\alpha_{BBB}\varsigma_{B}^{3}\right|$$

where $\alpha_{ijk}$ is given in Equation (9) of the main text. It should be remembered that there is no way to have *h* = 1 for $\Gamma_{ijk}^{\left(3\right)}$ of LAM mesophase. In BCC mesophase, forming a right triangle takes only 4 cases such as (${\vec{K}}_{1},-{\vec{K}}_{3},-{\vec{K}}_{6}$), (${\vec{K}}_{1},-{\vec{K}}_{4},-{\vec{K}}_{5}$), (${\vec{K}}_{2},-{\vec{K}}_{4},{\vec{K}}_{6}$), and (${\vec{K}}_{2},-{\vec{K}}_{3},{\vec{K}}_{5}$) in order to have *h* = 1.

The quartic form $A_{4}$ of the free energy functional is originally made of vertex functions of all possible combinations of indices *i*, *j*, *k*, and *l* as

(A8) $$\beta A_{4}=\frac{1}{4!}\sum_{{\vec{k}}_{i}\in \left\{\pm {\vec{K}}_{n}\right\}}\Gamma_{ijkl}\left({\vec{k}}_{1},{\vec{k}}_{2},{\vec{k}}_{3},{\vec{k}}_{4}\right)\frac{\varsigma_{i}\varsigma_{j}\varsigma_{k}\varsigma_{l}}{{\left(\sqrt{n}\right)}^{4}}e^{i\left(\varphi_{1}\left(i\right)+\varphi_{2}\left(j\right)+\varphi_{3}\left(k\right)+\varphi_{4}\left(l\right)\right)}$$

where ${\sum \vec{k}}_{i}=0$ must be satisfied by the integrand. The sums of phase angles are resolved by those of the second and third order terms. However, unlike $A_{3}$, the mesophase geometry is directly used for $A_{4}$. Still, the values of these sums are determined relative to $\sum \varphi_{l}(A)\equiv \varphi_{a}\left(A\right)+\varphi_{b}\left(A\right)+\varphi_{c}\left(A\right)+\varphi_{d}\left(A\right)$ for pure A correlations as follows:

(A9) $$\varphi_{a}\left(i\right)+\varphi_{b}\left(j\right)+\varphi_{c}\left(k\right)+\varphi_{d}\left(l\right)=\sum \varphi_{l}(A)-\pi \cdot c_{B}\left(ijkl\right)$$

where $c_{B}\left(ijkl\right)$ is the number of *B* among indices *i*, *j*, *k*, and *l*. Then, $A_{4}$ is obtained as

(A10) $$\beta A_{4}=\frac{1}{4!}\sum_{{\vec{k}}_{i}\in \left\{\pm {\vec{K}}_{n}\right\}}e^{i\left(\varphi_{a}\left(A\right)+\varphi_{b}\left(A\right)+\varphi_{c}\left(A\right)+\varphi_{d}\left(A\right)\right)}\Gamma_{ijkl}\left(h_{1},h_{2}\right)\frac{\varsigma_{i}\varsigma_{j}\varsigma_{k}\varsigma_{l}}{{\left(\sqrt{n}\right)}^{4}}e^{-i\cdot \pi c_{B}\left(ijkl\right)}$$

We need to count all the possible cases in accord with LAM, HEX, and BCC mesophases, which are given below in detail.

LAM:

In regards to the quartic form $A_{4}$ for lamellae, the only possible option to have the vanishing $\sum {\vec{k}}_{i}$ is ${\vec{k}}_{1}-{\vec{k}}_{1}+{\vec{k}}_{1}-{\vec{k}}_{1}=0$ or $\left(h_{1},h_{2}\right)=\left(0,0\right)$. This condition leads to $\sum \varphi_{i}\left(A\right)=\varphi_{a}\left(A\right)-\varphi_{a}\left(A\right)+\varphi_{a}\left(A\right)-\varphi_{a}\left(A\right)=0$. Then, the quartic form $A_{4}$ is expressed as

(A11) $$\beta A_{4}=\frac{3!}{4!}\Gamma_{ijkl}\left(0,0\right)\varsigma_{i}^{}\varsigma_{j}^{}\varsigma_{k}^{}\varsigma_{l}^{}e^{-i\pi \cdot c_{B}\left(ijkl\right)}$$

where all such cases are included.

HEX:

For the quartic form $A_{4}$ of HEX mesophase, the only possible sets of $\left(h_{1},h_{2}\right)$ are (0,0) and (0,1). After counting all such case, we have

(A12) $$\beta A_{4}=\frac{18}{4!{\left(\sqrt{3}\right)}^{4}}\left[\Gamma_{ijkl}\left(0,0\right)+4\Gamma_{ijkl}\left(0,1\right)\right]\varsigma_{i}^{}\varsigma_{j}^{}\varsigma_{k}^{}\varsigma_{l}^{}e^{-i\pi \cdot c_{B}\left(ijkl\right)}$$

It should be noted that $\left(h_{1},h_{2}\right)=\left(0,0\right)$ and $\left(0,1\right)$ respectively require one ${\vec{K}}_{a}$ ($\varphi_{a}-\varphi_{a}+\varphi_{a}-\varphi_{a}=0$ and two ${\vec{K}}_{j}$s forming either 60° or 120° between them ($\varphi_{b}-\varphi_{b}+\varphi_{c}-\varphi_{c}=0$).

BCC:

The quartic form $A_{4}$ of the free energy for BCC is given by

(A13) $$\begin{array}{l} \beta A_{4}=\frac{36}{4!{\left(\sqrt{6}\right)}^{4}}\left\{\Gamma_{ijkl}\left(0,0\right)e^{i\left(\varphi_{a}\left(A\right)-\varphi_{a}\left(A\right)+\varphi_{a}\left(A\right)-\varphi_{a}\left(A\right)\right)}+\right.\\ 8\Gamma_{ijkl}\left(0,1\right)e^{i\left(\varphi_{b}\left(A\right)-\varphi_{b}\left(A\right)+\varphi_{c}\left(A\right)-\varphi_{c}\left(A\right)\right)}+2\Gamma_{ijkl}\left(0,2\right)e^{i\left(\varphi_{d}\left(A\right)-\varphi_{d}\left(A\right)+\varphi_{e}\left(A\right)-\varphi_{e}\left(A\right)\right)}+\\ 2\Gamma_{ijkl}\left(1,2\right)\left.\left(e^{i\left(\varphi_{1}\left(A\right)+\varphi_{2}\left(A\right)-\varphi_{3}\left(A\right)-\varphi_{4}\left(A\right)\right)}+e^{i\left(\varphi_{1}\left(A\right)-\varphi_{2}\left(A\right)-\varphi_{5}\left(A\right)-\varphi_{6}\left(A\right)\right)}\right)\right\}\varsigma_{i}^{}\varsigma_{j}^{}\varsigma_{k}^{}\varsigma_{l}^{}e^{-i\pi \cdot c_{B}\left(ijkl\right)} \end{array}$$

There are only one ${\vec{K}}_{a}$ required for (0,0) contribution ($\varphi_{a}-\varphi_{a}+\varphi_{a}-\varphi_{a}=0$) and two ${\vec{K}}_{j}$’s required for (0,1) and (0,2) contributions ($\varphi_{b}-\varphi_{b}+\varphi_{c}-\varphi_{c}=0$) to $\beta F_{4}$. In the case of the (0,1) contribution, two ${\vec{K}}_{j}$’s form either 60° or 120° between them. In the other case of the (0,2) contribution, two ${\vec{K}}_{j}$’s should be selected to form the right angle. However, there are four ${\vec{K}}_{j}$’s necessary to describe (1,2) contribution to $\beta F_{4}$, as they are clearly expressed in Equation (A13). Using the result that $\varphi_{1}\left(A\right)-\varphi_{3}\left(A\right)-\varphi_{6}\left(A\right)=\varphi_{2}\left(A\right)-\varphi_{4}\left(A\right)+\varphi_{6}\left(A\right)=$ 0 or *π*, we have

(A14) $$\varphi_{1}\left(A\right)+\varphi_{2}\left(A\right)-\varphi_{3}\left(A\right)-\varphi_{4}\left(A\right)=\varphi_{1}\left(A\right)-\varphi_{3}\left(A\right)-\varphi_{6}\left(A\right)=0$$

or

(A15) $$\varphi_{1}\left(A\right)+\varphi_{2}\left(A\right)-\varphi_{3}\left(A\right)-\varphi_{4}\left(A\right)=\varphi_{1}\left(A\right)-\varphi_{3}\left(A\right)-\varphi_{6}\left(A\right)+\pi =2\pi$$

The same argument applies to the other set of phase angles in Equation (A13), because $\varphi_{1}\left(A\right)-\varphi_{4}\left(A\right)-\varphi_{5}\left(A\right)=\varphi_{2}\left(A\right)-\varphi_{4}\left(A\right)+\varphi_{6}\left(A\right)=$ 0 or *π*. Then, we have

(A16) $$\varphi_{1}\left(A\right)-\varphi_{2}\left(A\right)-\varphi_{5}\left(A\right)-\varphi_{6}\left(A\right)=\varphi_{1}\left(A\right)-\varphi_{4}\left(A\right)-\varphi_{5}\left(A\right)=0$$

or

(A17) $$\varphi_{1}\left(A\right)-\varphi_{2}\left(A\right)-\varphi_{5}\left(A\right)-\varphi_{6}\left(A\right)=\varphi_{1}\left(A\right)-\varphi_{4}\left(A\right)-\pi -\varphi_{5}\left(A\right)=0$$

Therefore, we have the final expression for the quartic form $A_{4}$ as

(A18) $$\beta A_{4}=\frac{36}{4!{\left(\sqrt{6}\right)}^{4}}\left[\Gamma_{ijkl}\left(0,0\right)+8\Gamma_{ijkl}\left(0,1\right)+2\Gamma_{ijkl}\left(0,2\right)+4\Gamma_{ijkl}\left(1,2\right)\right]\varsigma_{i}^{}\varsigma_{j}^{}\varsigma_{k}^{}\varsigma_{l}^{}e^{-i\pi \cdot c_{B}\left(ijkl\right)}$$

where $e^{-i\pi \cdot c_{B}\left(ijkl\right)}$ gives 1 or −1 depending on $c_{B}\left(ijkl\right)$ as before.

The Landau free energy is given by $\beta \Delta A=\beta A_{2}+\beta A_{3}+\beta A_{4}$ to have its final and general form in Equation (8) for each mesophase with the two amplitudes $\varsigma_{A}^{}$ and $\varsigma_{B}^{}$.

## Appendix B. Landau Free Energy through Transformation of Order Parameters

In this appendix, we provide the mathematical procedure to formulate our alternative Landau free energy in a more familiar form through the transformation of order parameters. The transformed order parameters, $\psi_{1}\left(\vec{r}\right)$ and $\psi_{2}\left(\vec{r}\right)$, are defined in the main text, where the former is a composite density profile in phase with A block and the latter indicates the negative fluctuations in free volume fraction. Upon this change of variables, the new vertex functions $\bar{\Gamma }$s are obtained from the original vertex functions Γs by Equation (15).

We will consider nanoscale mesophases, whose structures are defined by characteristic scattering vectors ${\vec{k}}_{1}\in \left\{\pm {\vec{K}}_{n}\right\}$. Those regular geometric morphologies are represented by $\psi_{1}$ as the sum of plane waves. The remaining $\psi_{2}$ is separated into two parts as $-\psi_{2}=-\psi_{2c}-\psi_{2i}$, where $-\psi_{2c}$ indicates the excess free volume at the interfaces between domains and $-\psi_{2i}$ represents the excess free volume in phase with the more compressible constituent. These order parameter parts are parametrized as given in the main text.

The quadratic form $A_{2}$ of the free energy expansion can be written as

(A19) $$\beta A_{2}\approx \frac{1}{2}\sum_{{\vec{k}}_{1}\in \left\{\pm {\vec{K}}_{n}\right\}}{\bar{\Gamma }}_{ij}^{}\left({\vec{k}}_{1},-{\vec{k}}_{1}\right)\psi_{i}({\vec{k}}_{1})\psi_{j}(-{\vec{k}}_{1})={\bar{\Gamma }}_{11}^{}\zeta_{1}^{2}+2{\bar{\Gamma }}_{12}^{}\zeta_{1}^{}\zeta_{2c}^{}e^{i\left(\varphi \left(1\right)-\varphi \left(2c\right)\right)}+{\bar{\Gamma }}_{22}^{}\zeta_{2c}^{2}+{\bar{\Gamma }}_{22}^{}\left(2q*\right)\zeta_{2i}^{2}$$

where the integral is approximated to the finite sum of the integrand at the characteristic wavevectors. Since ${\vec{k}}_{j}-\left(2{\vec{k}}_{j}\right)\neq 0$, there is no mixed term such as $\psi_{1}\psi_{2i}$.

We will discuss how to obtain higher-order terms in Equation (16) of the main text. This is where the present work takes a step forward with the deepened conception from our previous studies [45,46]. The cubic form $A_{3}$ is suggested in the following way. The fluctuations in free volume is considered to be small. Therefore, the only possible contributions in our 4-field theory are given by $\psi_{1}^{}\psi_{1}^{}\psi_{1}^{}$, $\psi_{1}^{}\psi_{1}^{}\psi_{2c}^{}$, and $\psi_{1}^{}\psi_{1}^{}\psi_{2i}^{}$as

(A20) $$\begin{array}{l} \beta A_{3}=\frac{1}{3!}\sum_{{\vec{k}}_{1}\in \left\{\pm {\vec{K}}_{n}\right\}}{\bar{\Gamma }}_{111}\left(1\right)\psi_{1}\left({\vec{k}}_{1}\right)\psi_{1}\left({\vec{k}}_{2}\right)\psi_{1}\left({\vec{k}}_{3}\right)+\frac{1}{3!}\sum_{{\vec{k}}_{1}\in \left\{\pm {\vec{K}}_{n}\right\}}\left({\bar{\Gamma }}_{112}\left(1\right)+{\bar{\Gamma }}_{121}\left(1\right)+{\bar{\Gamma }}_{211}\left(1\right)\right)\psi_{1}\left({\vec{k}}_{1}\right)\psi_{1}\left({\vec{k}}_{2}\right)\psi_{2c}\left({\vec{k}}_{3}\right)\\ +\frac{1}{3!}\sum_{{\vec{k}}_{1}\in \left\{\pm {\vec{K}}_{n}\right\}}\left({\bar{\Gamma }}_{112}\left(4\right)+{\bar{\Gamma }}_{121}\left(4\right)+{\bar{\Gamma }}_{211}\left(4\right)\right)\psi_{1}\left({\vec{k}}_{1}\right)\psi_{1}\left({\vec{k}}_{2}\right)\psi_{2i}\left(2,{\vec{k}}_{3}\right) \end{array}$$

The number *h* inside the bracket of ${\bar{\Gamma }}_{ijk}$ indicates the relative angles between ${\vec{k}}_{1}$, ${\vec{k}}_{2}$, and ${\vec{k}}_{3}$. Therefore, h=1 implies that those three vectors form a right triangle, whereas $h_{1}=4$ means that ${\vec{k}}_{1}={\vec{k}}_{2}$ and ${\vec{k}}_{3}=-2{\vec{k}}_{1}$. Equation (A20) is re-written with the parametrized order parameters as

(A21) $$\begin{array}{l} \beta A_{3}=\frac{1}{3!{\left(\sqrt{n}\right)}^{3}}\sum_{\left\{{\vec{K}}_{a},{\vec{K}}_{b},{\vec{K}}_{c}\right\}}{\bar{\Gamma }}_{111}\left(1\right)\zeta_{1}^{3}e^{i\left(\varphi_{a}\left(1\right)+\varphi_{b}\left(1\right)+\varphi_{c}\left(1\right)\right)}+\frac{1}{3!{\left(\sqrt{n}\right)}^{3}}\sum_{\left\{{\vec{K}}_{a},{\vec{K}}_{b},{\vec{K}}_{c}\right\}}\left(3{\bar{\Gamma }}_{112}\left(1\right)\right)\zeta_{1}^{2}\zeta_{2c}e^{i\left(\varphi_{a}\left(1\right)+\varphi_{b}\left(1\right)+\varphi_{c}\left(2c\right)\right)}\\ +\frac{1}{3!{\left(\sqrt{n}\right)}^{3}}\sum_{\left\{{\vec{K}}_{a},{\vec{K}}_{a},-2{\vec{K}}_{a}\right\}}\left(3{\bar{\Gamma }}_{112}\left(4\right)\right)\zeta_{1}^{2}\zeta_{2i}e^{i\left(\varphi_{a}\left(1\right)+\varphi_{a}\left(1\right)-\varphi \left(2i\right)\right)} \end{array}$$

where ${\bar{\Gamma }}_{112}={\bar{\Gamma }}_{121}={\bar{\Gamma }}_{211}$ is used above. In the same way, the quartic form $A_{4}$ of the free energy is only given by $\psi_{1}^{}\psi_{1}^{}\psi_{1}^{}\psi_{1}^{}$ as

(A22) $$\beta A_{4}=\frac{1}{4!}\sum_{{\vec{k}}_{i}\in \left\{\pm {\vec{K}}_{n}\right\}}{\bar{\Gamma }}_{1111}\left({\vec{k}}_{1},{\vec{k}}_{2},{\vec{k}}_{3},{\vec{k}}_{4}\right)\psi_{1}^{}\left({\vec{k}}_{1}\right)\psi_{1}^{}\left({\vec{k}}_{2}\right)\psi_{1}^{}\left({\vec{k}}_{3}\right)\psi_{1}^{}\left({\vec{k}}_{4}\right)=\frac{1}{4!{\left(\sqrt{n}\right)}^{4}}\sum_{\left\{{\vec{K}}_{a},{\vec{K}}_{b},{\vec{K}}_{c},{\vec{K}}_{d}\right\}}{\bar{\Gamma }}_{1111}\left(h_{1},h_{2}\right)\zeta_{1}^{4}e^{i\left(\varphi_{a}\left(1\right)+\varphi_{b}\left(1\right)+\varphi_{c}\left(1\right)+\varphi_{d}\left(1\right)\right)}$$

and other combination of the order parameters are ignored. In Equation (A22), the relative angles between ${\vec{k}}_{j}$s are described by $h_{1}$ and $h_{2}$ defined in the main text.

Let us try to minimize the free energy term by term starting with $A_{2}$. The A block is assumed to be less compressible. Then, ${\bar{\Gamma }}_{12}^{}\sim \Gamma_{11}^{}-\Gamma_{22}^{}<0$, which requires $\varphi \left(1\right)-\varphi \left(2c\right)=0$. The $\psi_{1}$ and $\psi_{2c}$ is then totally in phase with each other. The quadratic form $A_{2}$ is now written as

(A23) $$\beta A_{2}={\bar{\Gamma }}_{11}^{}\zeta_{1}^{2}+2{\bar{\Gamma }}_{12}^{}\zeta_{1}^{}\zeta_{2c}^{}+{\bar{\Gamma }}_{22}^{}\zeta_{2c}^{2}+{\bar{\Gamma }}_{22}^{}\left(2k*\right)\zeta_{2i}^{2}$$

Later, this relation between phase angles affects the sums of phase angles for the cubic and quartic forms of the free energy. The higher-order free energy terms are structure dependent. Therefore, we probe three classical structures including LAM, HEX, and BCC mesophases.

LAM:

Lamellae possess only one characteristic wave vector ${\vec{K}}_{1}$. Since it is only possible to form $\sum {\vec{k}}_{i}=0$ with ${\vec{k}}_{1}$, ${\vec{k}}_{1}$, and $-2{\vec{k}}_{1}$, the cubic form $A_{3}$ of the Landau free energy is solely given by $\psi_{1}^{}\psi_{1}^{}\psi_{2i}^{}$ as

(A24) $$\beta A_{3}=\frac{1}{3!{\left(\sqrt{1}\right)}^{3}}\sum_{{\vec{k}}_{i}\in \left\{\pm {\vec{K}}_{n}\right\}}\left(3{\bar{\Gamma }}_{112}\left(4\right)\right)\zeta_{1}^{2}\zeta_{2i}^{}e^{i\left(\varphi_{a}\left(1\right)+\varphi_{a}\left(1\right)-\varphi \left(2i\right)\right)}=\frac{2}{3!}\left(3{\bar{\Gamma }}_{112}\left(4\right)\right)\zeta_{1}^{2}\zeta_{2i}^{}$$

It is shown that ${\bar{\Gamma }}_{112}$ at *h* = 4 is negative, which requires that $\varphi \left(1\right)+\varphi \left(1\right)-\varphi \left(2i\right)=0$. The coefficient in Equation (A24) is $c_{4}^{LAM}$ in Equation (16) of the main text.

The quartic form $A_{4}$ of the Landau free energy for LAM is given by

(A25) $$\beta A_{4}=\frac{1}{4!{\left(\sqrt{1}\right)}^{4}}\sum_{{\vec{k}}_{i}\in \left\{\pm {\vec{K}}_{n}\right\}}{\bar{\Gamma }}_{1111}\left(0,0\right)\zeta_{1}^{4}e^{i\left(\varphi \left(1\right)-\varphi \left(1\right)+\varphi \left(1\right)-\varphi \left(1\right)\right)}=\frac{3!}{4!}{\bar{\Gamma }}_{1111}\left(0,0\right)\zeta_{1}^{4}=d_{n}^{LAM}\zeta_{1}^{4}$$

where only $\left(h_{1},h_{2}\right)$ = (0,0) is possible for our choice of wave vectors.

HEX:

The cubic form $A_{3}$ of the Landau free energy for HEX consists of

(A26) $$\begin{array}{l} \beta A_{3}=\frac{1}{3!{\left(\sqrt{3}\right)}^{3}}\sum_{{\vec{k}}_{i}\in \left\{\pm {\vec{K}}_{n}\right\}}{\bar{\Gamma }}_{111}\left(1\right)\zeta_{1}^{3}e^{i\left(\varphi_{1}\left(1\right)+\varphi_{2}\left(1\right)+\varphi_{3}\left(1\right)\right)}+\frac{1}{3!{\left(\sqrt{3}\right)}^{3}}\sum_{{\vec{k}}_{i}\in \left\{\pm {\vec{K}}_{n}\right\}}\left(3{\bar{\Gamma }}_{112}\left(1\right)\right)\zeta_{1}^{2}\zeta_{2c}^{}e^{i\left(\varphi_{1}\left(1\right)+\varphi_{2}\left(1\right)+\varphi_{3}\left(2c\right)\right)}\\ +\frac{1}{3!{\left(\sqrt{3}\right)}^{3}}\sum_{{\vec{k}}_{i}\in \left\{\pm {\vec{K}}_{n}\right\}}\left(3{\bar{\Gamma }}_{112}\left(4\right)\right)\zeta_{1}^{2}\zeta_{2i}^{}e^{i\left(\varphi_{a}\left(1\right)+\varphi_{a}\left(1\right)-\varphi \left(2i\right)\right)} \end{array}$$

For the first two parts, the situation is a bit complicated. It should be noticed that ${\bar{\Gamma }}_{112}\left(1\right)$’s and ${\bar{\Gamma }}_{112}\left(4\right)$’s are all negative. However, ${\bar{\Gamma }}_{111}(1)$ changes its sign, which leads to two different cases. In case that ${\bar{\Gamma }}_{12}^{}<0$ and ${\bar{\Gamma }}_{111}(1)<0$, $\varphi_{1}\left(1\right)+\varphi_{2}\left(1\right)+\varphi_{3}\left(1\right)=0$ and $\varphi_{1}\left(1\right)+\varphi_{2}\left(1\right)+\varphi_{3}\left(2c\right)=-\varphi_{3}\left(1\right)+\varphi_{3}\left(2c\right)=0$ in minimizing the free energy. Consider the opposite case that ${\bar{\Gamma }}_{12}^{}<0$ and ${\bar{\Gamma }}_{111}(1)>0$. If we choose $\varphi_{1}\left(1\right)+\varphi_{2}\left(1\right)+\varphi_{3}\left(1\right)=\pi$ for ${\bar{\Gamma }}_{111}(1)$ first, then $\varphi_{1}\left(1\right)+\varphi_{2}\left(1\right)+\varphi_{3}\left(2c\right)=-\varphi_{3}\left(1\right)+\pi +\varphi_{3}\left(2c\right)=\pi$ for ${\bar{\Gamma }}_{112}\left(1\right)$. If we choose $\varphi_{1}\left(1\right)+\varphi_{2}\left(1\right)+\varphi_{3}\left(2c\right)=0$ for ${\bar{\Gamma }}_{112}\left(1\right)$ first, then $\varphi_{1}\left(1\right)+\varphi_{2}\left(1\right)+\varphi_{3}\left(1\right)=-\varphi_{3}\left(2c\right)+\varphi_{3}\left(1\right)=0$ reversely for ${\bar{\Gamma }}_{111}(1)$. In all these cases, it is seen that $\varphi_{1}\left(1\right)+\varphi_{2}\left(1\right)+\varphi_{3}\left(2c\right)=\varphi_{1}\left(1\right)+\varphi_{2}\left(1\right)+\varphi_{3}\left(1\right)$. Owing to the negativity of ${\bar{\Gamma }}_{112}\left(4\right)$, it is gotten that $\varphi_{a}\left(1\right)+\varphi_{a}\left(1\right)-\varphi \left(2i\right)=0$. Summarizing all, $A_{3}$ should be as follows:

(A27) $$\begin{array}{l} \beta A_{3}=\frac{1}{3!{\left(\sqrt{3}\right)}^{3}}\sum_{\left\{{\vec{K}}_{a},{\vec{K}}_{b},{\vec{K}}_{c}\right\}}e^{i\left(\varphi_{a}\left(1\right)+\varphi_{b}\left(1\right)+\varphi_{c}\left(1\right)\right)}\cdot \left({\bar{\Gamma }}_{111}\left(1\right)\zeta_{1}^{3}+3{\bar{\Gamma }}_{112}\left(1\right)\zeta_{1}^{2}\zeta_{2c}\right)+\frac{6}{3!{\left(\sqrt{3}\right)}^{3}}\left(3{\bar{\Gamma }}_{112}\left(4\right)\right)\zeta_{1}^{2}\zeta_{2i}\\ =-\frac{12}{3!{\left(\sqrt{3}\right)}^{3}}\left|{\bar{\Gamma }}_{111}\left(1\right)\zeta_{1}^{3}+3{\bar{\Gamma }}_{112}\left(1\right)\zeta_{1}^{2}\zeta_{2c}\right|+\frac{6}{3!{\left(\sqrt{3}\right)}^{3}}\left(3{\bar{\Gamma }}_{112}\left(4\right)\right)\zeta_{1}^{2}\zeta_{2i} \end{array}$$

where the last expression is the outcome of minimizing $A_{3}$. The coefficients in Equation (A27) are $a_{n}^{HEX}$, $b_{1}^{HEX}$, and $c_{4}^{HEX}$ in Equation (16) of the main text. It should be emphasized that there is a chance to have ${\bar{\Gamma }}_{111}\left(1\right)\zeta_{1}^{}+3{\bar{\Gamma }}_{112}\left(1\right)\zeta_{2c}=0$, if ${\bar{\Gamma }}_{111}\left(1\right)>0$.

The quartic form $A_{4}$ is considered to contain only the contribution by $\psi_{1}^{}$ as

(A28) $$\begin{array}{l} \beta A_{4}=\frac{1}{4!}\sum_{{\vec{q}}_{i}\in \left\{\pm Q_{n}\right\}}{\bar{\Gamma }}_{1111}\left({\vec{q}}_{1},{\vec{q}}_{2},{\vec{q}}_{3},{\vec{q}}_{4}\right)\psi_{1}^{}\left({\vec{q}}_{1}\right)\psi_{1}^{}\left({\vec{q}}_{2}\right)\psi_{1}^{}\left({\vec{q}}_{3}\right)\psi_{1}^{}\left({\vec{q}}_{4}\right)\\ =\frac{1}{4!{\left(\sqrt{n}\right)}^{4}}\left\{\sum_{\left(0,0\right)}{\bar{\Gamma }}_{1111}\left(0,0\right)\right.\zeta_{1}^{4}e^{i\left(\varphi_{a}\left(1\right)-\varphi_{a}\left(1\right)+\varphi_{a}\left(1\right)-\varphi_{a}\left(1\right)\right)}+\frac{1}{{\left(\sqrt{n}\right)}^{4}}\sum_{\left(0,1\right)}{\bar{\Gamma }}_{1111}\left(0,1\right)\zeta_{1}^{4}\left.e^{i\left(\varphi_{b}\left(1\right)-\varphi_{b}\left(1\right)+\varphi_{c}\left(1\right)-\varphi_{c}\left(1\right)\right)}\right\} \end{array}$$

It can be seen that $\varphi_{a}\left(1\right)-\varphi_{a}\left(1\right)+\varphi_{a}\left(1\right)-\varphi_{a}\left(1\right)=0$ for $\left(h_{1},h_{2}\right)$ = (0,0) contribution and also $\varphi_{b}\left(1\right)-\varphi_{b}\left(1\right)+\varphi_{c}\left(1\right)-\varphi_{c}\left(1\right)=0$ for $\left(h_{1},h_{2}\right)$ = (0,1) contribution. Therefore, $A_{4}$ becomes

(A29) $$\beta A_{4}=\frac{18}{4!{\left(\sqrt{3}\right)}^{4}}\left[{\bar{\Gamma }}_{1111}\left(0,0\right)+4{\bar{\Gamma }}_{1111}\left(0,1\right)\right]\zeta_{1}^{4}=d_{n}^{HEX}\zeta_{1}^{4}$$

by counting all such cases for HEX mesophase.

BCC:

BCC mesophase requires the correct combinations from the six base vectors. The cubic form $A_{3}^{}$ is given as

(A30) $$\begin{array}{l} \beta A_{3}=\frac{1}{3!{\left(\sqrt{6}\right)}^{3}}\sum_{{\vec{k}}_{i}\in \left\{\pm {\vec{K}}_{n}\right\}}{\bar{\Gamma }}_{111}\left(1\right)\zeta_{1}^{3}\left\{e^{i\left(\varphi_{1}\left(1\right)-\varphi_{3}\left(1\right)-\varphi_{6}\left(1\right)\right)}+e^{i\left(\varphi_{1}\left(1\right)-\varphi_{4}\left(1\right)-\varphi_{5}\left(1\right)\right)}+e^{i\left(\varphi_{2}\left(1\right)-\varphi_{4}\left(1\right)+\varphi_{6}\left(1\right)\right)}+e^{i\left(\varphi_{2}\left(1\right)-\varphi_{3}\left(1\right)+\varphi_{5}\left(1\right)\right)}\right\}\\ +\frac{1}{3!{\left(\sqrt{6}\right)}^{3}}\sum_{{\vec{k}}_{i}\in \left\{\pm {\vec{K}}_{n}\right\}}3{\bar{\Gamma }}_{112}\left(1\right)\zeta_{1}^{3}\zeta_{2c}\left\{e^{i\left(\varphi_{1}\left(1\right)-\varphi_{3}\left(1\right)-\varphi_{6}\left(2c\right)\right)}+e^{i\left(\varphi_{1}\left(1\right)-\varphi_{4}\left(1\right)-\varphi_{5}\left(2c\right)\right)}+e^{i\left(\varphi_{2}\left(1\right)-\varphi_{4}\left(1\right)+\varphi_{6}\left(2c\right)\right)}+e^{i\left(\varphi_{2}\left(1\right)-\varphi_{3}\left(1\right)+\varphi_{5}\left(2c\right)\right)}\right\}\\ +\frac{1}{3!{\left(\sqrt{6}\right)}^{3}}\sum_{{\vec{k}}_{i}\in \left\{\pm {\vec{K}}_{n}\right\}}3{\bar{\Gamma }}_{112}\left(4\right)\zeta_{1}^{3}\zeta_{2i}e^{i\left(\varphi_{a}\left(1\right)+\varphi_{a}\left(1\right)-\varphi \left(2i\right)\right)} \end{array}$$

Regarding the first two parts, we need to do the same argument as that for HEX mesophase. In any cases, it is obtained that $\varphi_{a}\left(1\right)+\varphi_{b}\left(1\right)+\varphi_{c}\left(2c\right)=\varphi_{a}\left(1\right)+\varphi_{b}\left(1\right)+\varphi_{c}\left(1\right)$. The cubic form $A_{3}$ can then be written as

(A31) $$\begin{array}{l} \beta A_{3}=\frac{1}{3!{\left(\sqrt{6}\right)}^{3}}\sum_{\left\{{\vec{K}}_{a},{\vec{K}}_{b},{\vec{K}}_{c}\right\}}\left\{e^{i\left(\varphi_{1}\left(1\right)-\varphi_{3}\left(1\right)-\varphi_{6}\left(1\right)\right)}+e^{i\left(\varphi_{1}\left(1\right)-\varphi_{4}\left(1\right)-\varphi_{5}\left(1\right)\right)}+e^{i\left(\varphi_{2}\left(1\right)-\varphi_{4}\left(1\right)+\varphi_{6}\left(1\right)\right)}+e^{i\left(\varphi_{2}\left(1\right)-\varphi_{3}\left(1\right)+\varphi_{5}\left(1\right)\right)}\right\}[{\bar{\Gamma }}_{111}\left(1\right)\zeta_{1}^{3}\\ +3{\bar{\Gamma }}_{112}\left(1\right)\zeta_{1}^{2}\zeta_{2c}]+\frac{1}{3!{\left(\sqrt{6}\right)}^{3}}\sum_{\left\{{\vec{K}}_{a},{\vec{K}}_{b},{\vec{K}}_{c}\right\}}3{\bar{\Gamma }}_{112}\left(4\right)\zeta_{1}^{2}\zeta_{2i}e^{i\left(\varphi_{a}\left(1\right)+\varphi_{a}\left(1\right)-\varphi \left(2i\right)\right)} \end{array}$$

The sums of phase angles in the first bracket should go to either 0 or π simultaneously in order to minimize Equation (A31). The phase angles associated with ${\bar{\Gamma }}_{112}\left(4\right)$ sum up to 0 due to ${\bar{\Gamma }}_{112}\left(4\right)<0$. Summarizing all, we have the same type of formula for BCC as that for HEX as

(A32) $$\beta A_{3}=-\frac{48}{3!{\left(\sqrt{6}\right)}^{3}}\left|{\bar{\Gamma }}_{111}\left(1\right)\zeta_{1}^{3}+3{\bar{\Gamma }}_{112}\left(1\right)\zeta_{1}^{2}\zeta_{2c}^{}\right|+\frac{12}{3!{\left(\sqrt{6}\right)}^{3}}\cdot 3{\bar{\Gamma }}_{112}\left(4\right)\zeta_{1}^{2}\zeta_{2i}^{}$$

where the coefficients in Equation (A32) are $a_{n}^{BCC}$, $b_{1}^{BCC}$, and $c_{4}^{BCC}$ in Equation (16) of the main text.

The quartic form $A_{4}$ for BCC mesophase is given by

(A33) $$\begin{array}{l} \beta A_{4}=\frac{1}{4!{\left(\sqrt{6}\right)}^{4}}[\sum_{\left(0,0\right)}{\bar{\Gamma }}_{1111}\left(0,0\right)\zeta_{1}^{4}e^{i\left(\varphi_{a}\left(1\right)-\varphi_{a}\left(1\right)+\varphi_{a}\left(1\right)-\varphi_{a}\left(1\right)\right)}+\sum_{\left(0,1\right)}{\bar{\Gamma }}_{1111}\left(0,1\right)\zeta_{1}^{4}e^{i\left(\varphi_{b}\left(1\right)-\varphi_{b}\left(1\right)+\varphi_{c}\left(1\right)-\varphi_{c}\left(1\right)\right)}\\ +\sum_{\left(0,2\right)}{\bar{\Gamma }}_{1111}\left(0,2\right)\zeta_{1}^{4}e^{i\left(\varphi_{d}\left(1\right)-\varphi_{d}\left(1\right)+\varphi_{c}\left(1\right)-\varphi_{c}\left(1\right)\right)}+\sum_{\left(1,2\right)}{\bar{\Gamma }}_{1111}\left(1,2\right)\zeta_{1}^{4}\left\{e^{i\left(\varphi_{1}\left(1\right)+\varphi_{2}\left(1\right)-\varphi_{3}\left(1\right)-\varphi_{4}\left(1\right)\right)}+e^{i\left(\varphi_{1}\left(1\right)-\varphi_{2}\left(1\right)-\varphi_{5}\left(1\right)-\varphi_{6}\left(1\right)\right)}\right\} \end{array}$$

The sums of phase angles become zero for $\left(h_{1},h_{2}\right)$ = (0,0), (0,1), and (0,2). For $\left(h_{1},h_{2}\right)$ = (1,2) contributions, we provide a table to show all the results for those sums.

**Table A1:** Sum of phase angles associated with ${\bar{\Gamma }}_{1111}\left(1,2\right)$.

Case	*φ*_1_(1) + *φ*_2_(1) − *φ*_3_(1) − *φ*_4_(1) *^a^*	*φ*_1_(1) − *φ*_2_(1) − *φ*_5_(1) − *φ*_6_(1) *^b^*
$\varphi_{a}\left(1\right)+\varphi_{b}\left(1\right)+\varphi_{c}\left(1\right)=0$	$\begin{array}{l} \varphi_{1}\left(1\right)+\varphi_{2}\left(1\right)-\varphi_{3}\left(1\right)-\varphi_{4}\left(1\right)\\ =\varphi_{1}\left(1\right)-\varphi_{5}\left(1\right)-\varphi_{4}\left(1\right)=0 \end{array}$	$\begin{array}{l} \varphi_{1}\left(1\right)-\varphi_{2}\left(1\right)-\varphi_{5}\left(1\right)-\varphi_{6}\left(1\right)\\ =\varphi_{1}\left(1\right)-\varphi_{5}\left(1\right)-\varphi_{4}\left(1\right)=0 \end{array}$
$\varphi_{a}\left(1\right)+\varphi_{b}\left(1\right)+\varphi_{c}\left(1\right)=\pi$	$\begin{array}{l} \varphi_{1}\left(1\right)+\varphi_{2}\left(1\right)-\varphi_{3}\left(1\right)-\varphi_{4}\left(1\right)\\ =\varphi_{1}\left(1\right)+\pi -\varphi_{5}\left(1\right)-\varphi_{4}\left(1\right)=2\pi \end{array}$	$\begin{array}{l} \varphi_{1}\left(1\right)-\varphi_{2}\left(1\right)-\varphi_{5}\left(1\right)-\varphi_{6}\left(1\right)\\ =\varphi_{1}\left(1\right)-\varphi_{5}\left(1\right)-\varphi_{4}\left(1\right)-\pi =0 \end{array}$

*^a^* This sum is given from ${\vec{K}}_{1}+{\vec{K}}_{2}-{\vec{K}}_{3}-{\vec{K}}_{4}=0$ out of the six base vectors of BCC. *^b^* This sum is given from ${\vec{K}}_{1}-{\vec{K}}_{2}-{\vec{K}}_{5}-{\vec{K}}_{6}=0$

Using this table, $A_{4}$ is given as

(A34) $$\beta A_{4}=\frac{36}{4!{\left(\sqrt{6}\right)}^{4}}\left\{{\bar{\Gamma }}_{1111}\left(0,0\right)+8{\bar{\Gamma }}_{1111}\left(0,1\right)+2{\bar{\Gamma }}_{1111}\left(0,2\right)+4{\bar{\Gamma }}_{1111}\left(1,2\right)\right\}\zeta_{1}^{4}=d_{n}^{BCC}\zeta_{1}^{4}$$

where all such cases are corrected counted.

The Landau free energy is given by $\beta \Delta A=\beta A_{2}+\beta A_{3}+\beta A_{4}$ with the three amplitudes $\zeta_{1}^{}$, $\zeta_{2c}$, and $\zeta_{2i}$. Differentiating ΔA with respect to $\zeta_{2c}^{}$ and $\zeta_{2i}^{}$ and then nullifying those derivatives give Equation (23). Replacing such $\zeta_{2c}^{}$ and $\zeta_{2i}^{}$ into ΔA yields the final mathematical expression of our alternative Landau free energy in Equation (24).

## Appendix C. Free Energy Expansion through Fluctuation Correction in One-Loop Order

Diblock copolymers belong to Brazovskii universality class. In the mean-field picture, they possess their CP depending on disparity in self dispersion interactions. However, the effect of concentration fluctuations is known to destroy the mean-field CP to yield weak first order transition, as was revealed by Brazovskii [55]. A simplified fluctuation correction analysis adopted by Fredrickson and Helfand for incompressible diblock copolymers [56] is then applied to the compressible Landau energy, especially our alternative version in Equation (24). The free energy expansion βΔA is first divided by *η* as

(A35) $$\frac{\beta \Delta A}{\eta }=\left(\frac{{\bar{\Gamma }}_{11}^{}}{\eta }-\frac{{\bar{\Gamma }}_{12}^{2}}{\eta {\bar{\Gamma }}_{22}^{}}\right)\zeta_{1}^{2}-\left|\frac{a_{n}}{\eta }-\frac{b_{1}{\bar{\Gamma }}_{12}^{}}{\eta {\bar{\Gamma }}_{22}^{}}\right|\zeta_{1}^{3}+\frac{d_{n}}{\eta }\zeta_{1}^{4}$$

which is cast back to the integral expression as

(A36) $$\frac{\beta \Delta A}{\eta }\approx \sum_{n=2}^{4}\frac{1}{n!}\int {\bar{\Gamma }}_{n}^{\prime }\left({\vec{k}}_{1},\ldots ,{\vec{k}}_{n-1}\right)\cdot \psi_{1}\left({\vec{k}}_{1}\right)\ldots \psi_{n-1}\left({\vec{k}}_{n-1}\right)\psi_{n}\left(-\sum_{l=1}^{n-1}{\vec{k}}_{l}\right)$$

where

(A37) $${\bar{\Gamma }}_{2}^{\prime }\left(k^{*}\right)=\frac{{\bar{\Gamma }}_{11}^{}}{\eta }-\frac{{\bar{\Gamma }}_{12}^{2}}{\eta {\bar{\Gamma }}_{22}^{}}$$

and the remaining effective vertex coefficients, ${\bar{\Gamma }}_{3}^{\prime }$ and ${\bar{\Gamma }}_{4}^{\prime }$, are obtained in the corresponding fashion. The order parameter $\psi_{1}$ is rewritten with the more general order parameter ${\bar{\psi }}_{1}+{\tilde{\psi }}_{1}$, where ${\bar{\psi }}_{1}$ is the mean and ${\tilde{\psi }}_{1}$ the fluctuation part. An average external potential *Μ*, which is conjugate to ${\bar{\psi }}_{1}+{\tilde{\psi }}_{1}$, can be given from the functional differentiation of the free-energy expansion with respect to either ${\bar{\psi }}_{1}$ or ${\tilde{\psi }}_{1}$. After Brazovskii’s approximate closure relation [55] is employed, the inspection of *Μ* yields the following self-consistent equation for a function $S^{-1}$, which is the correction to $\Gamma_{2}^{\prime }$ owing to the fluctuation effects as

(A38) $${\bar{S}}^{-1}(k)={\bar{\Gamma }}_{2}^{\prime }(k)+{\bar{\Gamma }}_{4}^{\prime }(k^{*}){\bar{\psi }}^{2}+\frac{1}{2}{\bar{\Gamma }}_{4}^{\prime }(k^{*})\cdot \int d{\vec{k}}_{1}\bar{S}({\vec{k}}_{1})$$

Because of the profound minimum of ${\bar{\Gamma }}_{2}^{\prime }$ at $k^{*}$, it is expanded around $k^{*}$ up to the quadratic order as ${\bar{\Gamma }}_{2}^{\prime }(k)={\bar{\Gamma }}_{2}^{\prime }(k^{*})+c{\left(k-k^{*}\right)}^{2}$, where the symbol *c* indicates $c=1/2\cdot \partial^{2}{\left.{\bar{\Gamma }}_{2}^{\prime }/\partial k^{2}\right)}_{k^{*}}$. This expression for ${\bar{\Gamma }}_{2}^{\prime }$ is put into Equation (A38) to yield ${\bar{S}}^{-1}(k)={\bar{S}}^{-1}(k^{*})+c{\left(k-k^{*}\right)}^{2}$, which helps us to evaluate an ultraviolet divergent integral $\int d{\vec{k}}_{1}\bar{S}({\vec{k}}_{1})$ as

(A39) $$\int d{\vec{k}}_{1}\bar{S}({\vec{k}}_{1})=(3x^{*}/N_{c}\pi )/\sqrt{{\bar{S}}^{-1}(k^{*})\cdot \tilde{c}}$$

The new symbol $\tilde{c}$ is given by $\tilde{c}=N_{c}x^{*}/3\cdot \partial^{2}{\left.{\bar{\Gamma }}_{2}^{\prime }/\partial x^{2}\right)}_{x^{*}}$, where *x* = $k^{2}R_{G}^{2}$ as before. The corresponding $x^{*}$ is then evaluated at $k^{*}$. The ${\bar{S}}^{-1}$ at $k^{*}$ then becomes

(A40) $${\bar{S}}^{-1}(k^{*})={\bar{\Gamma }}_{2}^{\prime }(k^{*})+{\bar{\Gamma }}_{4}^{\prime }(k^{*})\zeta^{2}+\frac{{\bar{\Gamma }}_{4}^{\prime }(k^{*})\cdot 3x^{*}/2\pi }{N_{c}\sqrt{{\bar{S}}^{-1}(k^{*})\cdot \tilde{c}}}$$

where ζ is the amplitude parameter of ${\bar{\psi }}_{1}$. The desired free energy with the inclusion of fluctuation correction is now given from the integration of the approximate expression for *Μ* as

(A41) $$\frac{\beta \Delta A}{\eta }=\frac{1}{2{\bar{\Gamma }}_{4}^{\prime }}\left({\bar{S}}^{-2}(k^{*})-{\bar{S}}_{D}^{-2}(k^{*})\right)+\frac{3x^{*}/2\pi }{N_{c}\sqrt{\tilde{c}}}\left(\sqrt{{\bar{S}}^{-1}(k^{*})}-\sqrt{{\bar{S}}_{D}^{-1}(k^{*})}\right)-\left|\frac{a_{n}}{\eta }-\frac{b_{1}{\bar{\Gamma }}_{12}^{}}{\eta {\bar{\Gamma }}_{22}^{}}\right|\zeta^{3}+\left(\frac{d_{n}}{\eta }-\frac{{\bar{\Gamma }}_{4}^{\prime }}{2}\right)\zeta^{4}$$

where ${\bar{S}}_{D}^{-1}$ represents ${\bar{S}}^{-1}$ in the disordered state, and can thus be obtained if ζ in Equation (A40) is taken to be zero.
