# Supplementary material for: Effect of Disparity in Self Dispersion Interactions on Phase Behaviors of Molten A-b-B Diblock Copolymers
Source: Polymers (Basel). 2022 Dec 21;15(1):30. doi: 10.3390/polym15010030 (PMC9824038; doi:10.3390/polym15010030)
Supplement: Supplementary file 1 [file polymers-15-00030-s001.zip › polymers-2035722-supplementary.pdf]

polymers

MDPI

Supplementary Material

Effect of Disparity in Self Dispersion Interactions on Phase Behaviors of Molten A-b-B Diblock Copolymers

Xinyue Zhang, Mingge Zhao and Junhan Cho *

Department of Polymer Science & Engineering, Dankook University, 152 Jukjeon-ro, Suji-gu, Yongin, Gyeonggi-do 16890, Republic of Korea
* Correspondence: jhcho@dankook.ac.kr; Tel.: +82-31-8005-3586

I. Vertex Coefficients for PS-b-PBD and PS-b-PVME at selected compositions

Table S1. Characteristic squared wavenumber x* at spinodals for PS-b-PBD and PS-b-PVME melts listed at selected compositions ϕ_A s.

ϕ_A [a]	x* at spinodals	
	**PS-b-PBD**	**PS-b-PVME**
0.1	5.5515	5.5485
0.2	4.4715	4.4685
0.3	4.0395	4.0380
0.4	3.8430	3.8430
0.5	3.7845	3.7845
0.6	3.8430	3.8430
0.7	4.0395	4.0410
0.8	4.4715	4.4730
0.9	5.5515	5.5545

[a] A implies PS block.

Table S2. Quadratic vertex coefficients Γ_{ij} s for A-b-B with $\overline{\varepsilon}_{AA} = \overline{\varepsilon}_{BB}$ at the indicated compositions ϕ_A s.

ϕ_A	A-b-B [a]		
	Γ_{AA}	Γ_{AB}	Γ_{BB}
0.1	315.959	315.820	315.680
0.2	96.502	96.425	96.348
0.3	52.997	52.955	52.913
0.4	38.946	38.927	38.908
0.5	35.358	35.358	35.358
0.6	38.908	38.927	38.946
0.7	52.913	52.955	52.997
0.8	96.348	96.425	96.502
0.9	315.680	315.820	315.959

Table S3. Cubic vertex coefficients $\eta^2 N_c \Gamma_{ijk}$ s for A-b-B at the indicated compositions ϕ_A s.

ϕ_A	$\eta^2 N_c \Gamma_{ijk}$ [a]			
	$\eta^2 N_c \Gamma_{AAA}$	$\eta^2 N_c \Gamma_{AAB}$	$\eta^2 N_c \Gamma_{ABB}$	$\eta^2 N_c \Gamma_{BBB}$
0.1	-1315.699	1.233	5.216	-6.731
0.2	-192.764	1.627	2.688	-7.263
0.3	-65.597	1.487	1.916	-8.984
0.4	-31.550	1.418	1.586	-12.232
0.5	-18.415	1.444	1.444	-18.415
0.6	-12.232	1.586	1.418	-31.550
0.7	-8.984	1.916	1.487	-65.597
0.8	-7.263	2.688	1.627	-192.764
0.9	-6.731	5.216	1.233	-1315.699

[a] These values are determined at their respective spinodal points.

Table S4. Quadratic vertex coefficients Γ_{ij} s for PS-b-PBD and PS-b-PVME at the indicated compositions ϕ_A s.

ϕ_A	**PS-b-PBD** [a]			**PS-b-PVME** [a]		
	Γ_{AA}	Γ_{AB}	Γ_{BB}	Γ_{AA}	Γ_{AB}	Γ_{BB}
0.1	313.410	313.445	313.479	24.449	25.472	26.538
0.2	95.666	95.721	95.776	39.788	40.992	42.232
0.3	52.520	52.586	52.652	42.515	43.730	44.980
0.4	38.590	38.668	38.747	43.009	44.213	45.449
0.5	35.044	35.138	35.232	42.672	43.855	45.071
0.6	38.628	38.744	38.860	41.734	42.892	44.082
0.7	52.652	52.802	52.952	39.898	41.022	42.178
0.8	96.157	96.364	96.572	35.569	36.633	37.729
0.9	315.907	316.219	316.532	17.474	18.288	19.140

[a] These values are determined at their respective spinodal points.

Table S5. Quartic vertex coefficients $\eta^3 N_c \delta^{BCC}_{ijkl} / c_{BCC}$ s in Equation (12) for BCC-forming PS-b-PBD at the indicated ϕ_A s.

ϕ_A	**comment** [a b]				
	$\frac{\eta^3 N_c \delta^{BCC}_{AAAA}}{c_{BCC}}$	$\frac{\eta^3 N_c \delta^{BCC}_{AAAB}}{c_{BCC}}$	$\frac{\eta^3}{3c_{BCC}} N_c \left(\delta^{BCC}_{AABB} + \delta^{BCC}_{ABAB} + \delta^{BCC}_{ABBA}\right)$	$\frac{\eta^3 N_c \delta^{BCC}_{ABBB}}{c_{BCC}}$	$\frac{\eta^3 N_c \delta^{BCC}_{BBBB}}{c_{BCC}}$
0.1	422669.520	-20.100	-53.241	137.637	292.611
0.2	31837.194	22.243	-43.249	65.823	344.358
0.3	7388.133	26.265	-35.172	44.189	473.868
0.4	2722.107	27.625	-31.273	34.675	734.811
0.5	1298.298	29.953	-30.121	29.953	1298.298
0.6	734.811	34.675	-31.273	27.625	2722.107
0.7	473.868	44.189	-35.172	26.265	7388.133
0.8	344.358	65.823	-43.249	22.243	31837.194
0.9	292.611	137.637	-53.241	-20.100	422669.520

[a] These values are determined at their respective spinodal points. [b] $c_{BCC} = 36/4!\left(\sqrt{6}\right)^4$.

Table S6. Quartic vertex coefficients $\eta^3 N_c \delta_{ijkl}^{HEX} / c_{HEX}$ s in Equation (11) for cylinder-forming PS-b-PBD at the indicated ϕ_A s.

ϕ_A	comment [a b]				
	$\frac{\eta^3 N_c \delta_{AAAA}^{HEX}}{c_{HEX}}$	$\frac{\eta^3 N_c \delta_{AAAB}^{HEX}}{c_{HEX}}$	$\frac{\eta^3}{3c_{HEX}} N_c \left(\delta_{AABB}^{HEX} + \delta_{ABAB}^{HEX} + \delta_{ABBA}^{HEX}\right)$	$\frac{\eta^3 N_c \delta_{ABBB}^{HEX}}{c_{HEX}}$	$\frac{\eta^3 N_c \delta_{BBBB}^{HEX}}{c_{HEX}}$
0.1	140767.628	-34.880	-25.291	43.998	97.187
0.2	10588.676	-1.484	-18.365	20.272	114.128
0.3	2453.829	4.148	-14.553	13.060	156.938
0.4	902.995	6.221	-12.813	9.717	243.378
0.5	430.267	7.757	-12.307	7.757	430.267
0.6	243.378	9.717	-12.813	6.221	902.995
0.7	156.938	13.060	-14.553	4.148	2453.829
0.8	114.128	20.272	-18.365	-1.484	10588.676
0.9	97.187	43.998	-25.291	-34.880	140767.628

[a] These values are determined at their respective spinodal points. [b] $c_{HEX} = 18 / 4!\left(\sqrt{3}\right)^4$.

Table S7. Quartic vertex coefficients $\eta^3 N_c \delta_{ijkl}^{LAM} / c_{LAM}$ s in Equation (10) for lamella-forming PS-b-PBD at the indicated ϕ_A s.

ϕ_A	comment [a b]				
	$\frac{\eta^3 N_c \delta_{AAAA}^{LAM}}{c_{LAM}}$	$\frac{\eta^3 N_c \delta_{AAAB}^{LAM}}{c_{LAM}}$	$\frac{\eta^3}{3c_{LAM}} N_c \left(\delta_{AABB}^{LAM} + \delta_{ABAB}^{LAM} + \delta_{ABBA}^{LAM}\right)$	$\frac{\eta^3 N_c \delta_{ABBB}^{LAM}}{c_{LAM}}$	$\frac{\eta^3 N_c \delta_{BBBB}^{LAM}}{c_{LAM}}$
0.1	28102.894	-17.928	-7.910	8.096	19.307
0.2	2108.194	-3.656	-5.124	3.446	22.585
0.3	487.280	-0.878	-3.935	2.008	31.015
0.4	178.920	0.150	-3.421	1.278	48.098
0.5	85.113	0.743	-3.274	0.743	85.113
0.6	48.098	1.278	-3.421	0.150	178.920
0.7	31.015	2.008	-3.935	-0.878	487.280
0.8	22.585	3.446	-5.124	-3.656	2108.194
0.9	19.307	8.096	-7.910	-17.928	28102.894

[a] These values are determined at their respective spinodal points. [b] $c_{LAM} = 1/4$.
